# How Can Transcranial Magnetic Stimulation Be Used to Modulate Episodic Memory?: A Systematic Review and Meta-Analysis

**DOI:** 10.3389/fpsyg.2019.00993

**Published:** 2019-06-13

**Authors:** Nicholas Yeh, Nathan S. Rose

**Affiliations:** Department of Psychology, University of Notre Dame, Notre Dame, IN, United States

**Keywords:** non-invasive brain stimulation, transcranial magnetic stimulation, repetitive TMS, theta-burst stimulation, episodic memory, recall, recognition

## Abstract

A systematic review and meta-analysis were conducted to synthesize the existing literature on how transcranial magnetic stimulation (TMS) has been used to modulate episodic memory. Given the numerous parameters of TMS protocols and experimental design characteristics that can be manipulated, a mechanistic understanding of how changes in the combination of parameters (e.g., frequency, timing, intensity, targeted brain region, memory task) modulate episodic memory is needed. To address this, we reviewed 59 studies and conducted a meta-analysis on 245 effect sizes from 37 articles on healthy younger adults (*N* = 1,061). Analyses revealed generally more beneficial effects of 1-Hz rTMS vs. other frequencies on episodic memory. Moderation analyses revealed complex interactions as online 20-Hz rTMS protocols led to negative effects, while offline 20-Hz rTMS led to enhancing effects. There was also an interaction between stimulation intensity and frequency as 20-Hz rTMS had more negative effects when applied below- vs. at-motor threshold. Conversely, 1-Hz rTMS had more beneficial effects than other frequencies when applied below- vs. at- or above-motor threshold. No reliable aggregate or hypothesized interactions were found when assessing stimulation site (frontal vs. parietal cortex, left vs. right hemisphere), stimulated memory process (during encoding vs. retrieval), the type of retrieval (associative/recollection vs. item/familiarity), or the type of control comparison (active vs. sham or no TMS) on episodic memory. However, there is insufficient data to make strong inference based on the lack of aggregate or two-way interactions between these factors, or to assess more complex (e.g., 3-way) interactions. We reviewed the effects on other populations (healthy older adults and clinical populations), but systematic comparison of parameters was also prevented due to insufficient data. A database of parameters and effects sizes is available as an open source repository so that data from studies can be continuously accumulated in order to facilitate future meta-analysis. In conclusion, modulating episodic memory relies on complex interactions among the numerous moderator variables that can be manipulated. Therefore, rigorous, systematic comparisons need to be further investigated as the body of literature grows in order to fully understand the combination of parameters that lead to enhancing, detrimental or null effects on episodic memory.

Over the past 30 years, transcranial magnetic stimulation (TMS) has enabled researchers to move beyond correlational research and address causal relations between brain and behavior in humans. While powerful, the mechanisms of TMS and the resulting outcomes on higher order cognitive functioning such as episodic memory are complex and mired by differences across studies. As the body of research on TMS continues to emerge, rigorous, and systematic comparisons need to be investigated to better understand the moderating effects of the various parameters of TMS that can be manipulated. To this aim, we first reviewed studies that have used repetitive TMS (rTMS) in attempt to modulate episodic memory. Next, we conducted a meta-analysis to bridge the gap in understanding of aspects of rTMS protocols that lead to memory enhancement or impairment. Lastly, we reviewed and compared the limited number of studies that have used rTMS to modulate episodic memory in healthy older adults and those who suffer from clinical disorders.

In a systematic review, we focus on examining the effects of rTMS on episodic memory, which entails vividly remembering past events along with their spatial, temporal, and/or source details (Tulving, 2002). A full understanding of the mechanisms underlying cognitive processes in episodic memory is critical for developing effective interventions to target memory deficits that occur in healthy aging and in those with clinical disorders. However, the neural substrates of episodic memory are difficult to directly observe and manipulate and the field has generally relied on neuroimaging techniques that have provided important, albeit correlational, findings linking neural activity in specific brain regions with episodic memory processes. The introduction of TMS techniques has made it possible to make more causal claims about the role of specific brain regions (and functionally coupled regions) in specific processes by temporarily and reversibly activating neuronal firing in targeted cortical regions. Such exogenous stimulation can induce the activation of specific neural circuits and/or perturb or disrupt endogenous neural activity in targeted and functionally connected areas, and then allows for investigation of the resulting effects on behavior, as well as on associated neural signals when TMS is coupled with simultaneous neuroimaging. Therefore, it is critical to both understand the ways in which varying rTMS parameters can reveal the role of neurocognitive processes in supporting episodic memory, and how rTMS may be used to enhance or impair episodic memory functioning (for early reviews, see Grafman and Wassermann, 1998; Manenti et al., 2012). Until now, there has been no systematic review or meta-analysis on the effects of rTMS on episodic memory.

## Principles of rTMS and Episodic Memory That Are Important for Researchers to Understand

When conducted properly, TMS is a relatively safe and painless technique that utilizes electromagnetism to induce current in the brain that can cause neurons to fire (Rossi et al., 2009). During TMS, an electrical current passes through a coil of insulated wiring and produces brief magnetic “pulses” to induce neural firing. These transitory currents cause rapid depolarization (i.e., action potentials) of neurons beneath the coil and can modulate cortical excitability with a relatively high level of spatial and temporal precision (Wagner et al., 2009). Importantly, through polysynaptic connections, action potentials can propagate to distal brain regions that are functionally connected at the time of stimulation.

The application of TMS protocols can be roughly classified with respect to the amount of time between pulses. For example, single and paired pulse procedures vs. repetitive TMS reflect differences in the time between delivering pulses. rTMS has been implemented to modulate cortical excitability with the intent to either enhance or attenuate subsequent cognitive and behavioral outcomes (Pascual-Leone et al., 1998). Stimulation parameters such as frequency, intensity, and stimulation timing have been shown to be critical in achieving the desired response. Multimodal techniques (i.e., TMS-EEG, TMS-fMRI) and machine learning analyses (i.e., multivariate pattern analysis) have revealed the importance of understanding the relationship between the anatomical and functional brain regions being targeted and the cognitive representations and processes that are coded in the neural signals and may be manipulated by stimulation. In other words, stimulation parameters interact with ongoing patterns of neural activity to drive changes in cognition and behavior (Silvanto et al., 2008; Romei et al., 2016). TMS techniques have grown in popularity due the feasibility of non-invasively modulating brain activity to study the role of specific brain regions in cognitive processes and modify behavior in awake, behaving humans. The techniques have far reaching implications within the field of memory for the development of treatments for people suffering from memory deficits due to normal aging or neurodegenerative disorders. As reviewed below, findings are often quite mixed and variable between individuals; thus, increased understanding of the most proficient rTMS protocols for effectively enhancing episodic memory is critical in order to achieve translational applications in clinical settings.

### Episodic Memory

Memory is not an isomorphic construct and, of the various forms of memory, episodic memory is the form that is absolutely critical for maintaining one's self-identity. According to Tulving (2002), episodic memory is the type of declarative memory that represents knowledge of particular events that one has experienced as well as their contextual acquisition (e.g., autobiographical memory), such as when and where an event occurred (e.g., who you met at a dinner party last week). It is measured by retrieval accuracy on declarative memory tests such as recall and recognition. Episodic memory requires and extends a related type of declarative memory, i.e., semantic memory or one's general knowledge of the world. Semantic memory involves retrieving knowledge (i.e., facts, concepts, meaning) without the accompanying contextual information (e.g., knowing how many planets are in the solar system, but without remembering in vivid detail when or where this was learned). The critical role of the hippocampus and surrounding structures of the medial temporal lobes (MTL) in the encoding, consolidation, and retrieval of episodic memories has been highlighted by striking cases of amnesia following either surgical resection (as in patient HM), disease (as in Clive Wearing), or brain trauma (as in patient KC), and have been elucidated by decades of animal/behavioral, neuropsychological, neuroimaging, and neurostimulation research (for reviews see, Moscovitch et al., 2016; Eichenbaum, 2017).

The hippocampus alone does not support episodic memory, however. In order for an event to be remembered, it must first be encoded through some combination of perceptual (e.g., visual, acoustic), affective, contextual, and semantic processes that integrate an event with its contextual associations to form episodic memories. Additionally, executive control processes are thought to facilitate the multi-modal binding of features and associations for episodic memory representations and are thought to be mediated by regions of the prefrontal cortex (PFC) and parietal cortex (PC; Buckner et al., 1999). Encoding and retrieval processes are inherently intertwined. Once encoded, successful retrieval demonstrates access to a memory, which also serves as a measure for successful encoding. For example, the encoding specificity principle (Tulving and Thomson, 1973) and related concept of “transfer-appropriate-processing” (Morris et al., 1977; Blaxton, 1989) emphasize the role of a reinstatement of the encoding context during retrieval for episodic memory. At the neural level, context reinstatement is operationalized by the degree of overlap in neural activity elicited during encoding and retrieval, and functional neuroimaging studies have revealed the involvement of regions in the prefrontal (Prince, 2005), medial temporal (Rugg and Vilberg, 2013), and parietal cortices (Wagner et al., 2005) (for review, see Craik and Rose, 2012).

### The Role of Encoding and Retrieval

Although encoding and retrieval are intertwined, there are important differences in the neural correlates of successful episodic encoding and retrieval. For example, there is often a hemispheric asymmetry in left vs. right PFC activation during encoding vs. retrieval, depending on the task stimuli, familiarity, and the cognitive processes engaged (see below). With regards to rTMS, it is important to note that the neural substrates of episodic memory are impacted by the underlying state of activation in a stimulated brain region that is driven by the task demands and stimuli (e.g., words vs. pictures; Miniussi et al., 2013; Romei et al., 2016).

### Verbal Stimuli

It is generally theorized that, in younger adults, the left PFC is critical for encoding, while the right PFC is to be preferentially involved in retrieval (Habib et al., 2003). This is known as the hemispheric encoding-retrieval asymmetry (HERA) model, which has been supported by some evidence from rTMS findings (Rossi et al., 2004). However, the role of the left and right PFC in episodic memory may also vary due to numerous factors, such as task stimuli. For example, in two studies, left DLPFC stimulation impaired memory for verbal information (Rami et al., 2003), while right DLPFC stimulation impaired memory for non-verbal stimuli (Epstein et al., 2002; Floel et al., 2004). However, differences between the parameters of stimulation such as frequency and intensity across studies complicate direct comparison.

### Stimulus Familiarity

The effects of rTMS on episodic memory has also been shown to vary with an individual's familiarity with the type of information that is to be remembered. For example, online 20 Hz rTMS to the left DLPFC during retrieval impaired novel compared to familiar information (Sandrini et al., 2003). Analogously, rTMS has been shown to preferentially affect memory for contextual information of studied associations. For example, online 10 Hz rTMS to the left inferior frontal gyrus (IFG) during encoding of novel faces paired with a “context” stimulus (e.g., the word “lawyer”) impaired subsequent recognition of faces compared to faces studied without a context stimulus (i.e., faces studied with “no context”; Feurra et al., 2010). Thus, effects of rTMS on episodic memory likely depends not only on the specific rTMS parameters (e.g., frequency, intensity, stimulation timing, or target site), but also on the cognitive processes that are tapped by the type of information presented or the way in which it is to be remembered.

### Cognitive Processes Engaged

In a similar vein, the effects of rTMS on episodic memory likely also depends on the cognitive strategies involved during encoding and/or retrieval (Manenti et al., 2010b, 2012; Hawco et al., 2013). For example, Innocenti et al. (2010) had individuals engage in “deep” (semantic) or “shallow” (perceptual) levels of processing while encoding words and applying online 10 Hz rTMS to either the left or right DLPFC. There were dissociable effects on recognition of words that were deeply or shallowly encoded, with left DLPFC stimulation abolishing the typical benefit of deep processing on memory (Innocenti et al., 2010). Relatedly, in a face-name pair encoding task, participants received fMRI-guided 10 Hz rTMS to either the left or right DLPFC during retrieval. After subdividing performance for those who reported using a retrieval strategy from those who reported using no retrieval strategy, it was concluded that right DLPFC stimulation disrupted retrieval for strategy users, while left DLPFC stimulation disrupted retrieval for non-strategy users (Manenti et al., 2010b).

Taken together, the effects of rTMS on episodic memory likely depend not only on the specific parameters of the protocol, but also on the brain state and cognitive processes engaged at the time of stimulation. This brain-state dependence is consistent with research that has used TMS in a wide variety of domains that has shown that the effects of TMS to a targeted region interacts with the ongoing, endogenous neural activity in that region and functionally coupled regions at the time of stimulation. Thus, while patterns are complicated across conditions, this principle enables researchers to investigate the causal role of the targeted and functionally coupled regions in specific neurocognitive mechanisms through the systematic manipulation of different task conditions (for review see, Romei et al., 2016).

### The Role of Retrieval Processes

According to dual-process models of episodic memory, the cognitive processes engaged during retrieval can be supported by two separate processes (i.e., familiarity and recollection). A central theme of dual-process models is that previously studied information supports recognition by being generally more “familiar” than new/non-studied information. A distinct retrieval process, “recollection,” can further facilitate recognition of previously studied information through the retrieval of associated spatial, temporal, or contextual information (Yonelinas, 2002). The remember/know procedure is commonly implemented to examine differences in the nature or subjective experience of retrieval. Specifically, during a recognition task, participants indicate if the decision to respond that a presented item was previously seen (i.e., old) was based on recollection of the encoding details or based on a feeling of familiarity; they are instructed to respond with “remember” or “know,” respectively, (Tulving, 1985).

rTMS enables the ability to causally investigate the underlying neural substrates of familiarity and recollection processes. For example, Turriziani et al. (2008) applied online 20 Hz rTMS to the left or right DLPFC during either encoding or retrieval (i.e., remember/know paradigm) of faces. They found that recollection (remember responses) was impaired following right compared to left DLPFC stimulation during encoding. However, familiarity was impaired following left and right DLPFC stimulation during encoding. These findings suggest that both the left and right DLPFC support familiarity and recollective retrieval processes, but the left vs. right DLPFC may contribute differentially depending on the type of retrieval process engaged.

### The Role of Cortical Regions

The traditional view of episodic memory has focused on the hippocampus and its role in binding specific details of an experience (such as what events have occurred) with when (temporal), where (spatial), or how (source) the events occurred (Tulving, 1985, 2002). A large body of work on patient H.M., who had extensive surgical resection of the bilateral hippocampi and surrounding structures of the MTL in young adulthood, revealed the importance of the hippocampus in the formation of new episodic memories (Squire, 2009). However, inferences from brain lesion studies are often confounded by compensatory reorganization mechanisms (e.g., plasticity) that occur following brain injury and/or damage that is not isolated to specific brain regions. Alternatively, TMS can be used to causally modulate neural activity in healthy brain regions and networks at the precise time that a hypothesized cognitive operation is thought to occur; it affords a direct test of how specific brain regions contribute to episodic memory. However, the limited spatial ability to target deep regions in the MTL has led to substantial focus on targeting neocortical regions. The combination of TMS with neuroimaging (e.g., fMRI, EEG) and functional connectivity analyses provides a promising route to understanding brain network interactions, and has enabled further understanding of how TMS related changes in local neural activity propagate through polysynaptic connections to distal regions that are functionally connected at the time of stimulation (Shafi et al., 2012). For example, several studies have shown effects on episodic memory from targeting nodes in neocortical networks in prefrontal cortex or parietal cortex that are functionally connected with the hippocampus (e.g., Wang et al., 2014).

### Prefrontal Cortex

The PFC in particular has received substantial attention as a stimulation site in rTMS protocols. For example, a fMRI-localized rTMS study targeted the left inferior prefrontal cortex (LIPFC) online during a word-pair encoding task with 7 Hz rTMS. In comparison to two active control conditions (i.e., RIPFC, left superior partial cortex; LPC), stimulation of the LIPFC enhanced subsequent memory on an immediate word recognition test. The authors suggested that this causal link between LIPFC and episodic memory may have been driven by LIPFC stimulation triggering elaborative processing of word pairs that led to more distinctive memory cues (Köhler et al., 2004). One important design aspect of this study that we will briefly return to later is the fMRI-localization targeting procedure that accounts for individual differences in the structure and function of stimulated brain regions for more accurate targeting of regions of interest in a specific task.

### Parietal Cortex

While PFC-MTL interactions are a cornerstone of neurocognitive models of episodic memory, it is also clear that there is extensive, often task-dependent recruitment of distributed networks of areas that process sensory, semantic, or emotional information to support various aspects of episodic memory (Ciaramelli et al., 2008; Cabeza and Moscovitch, 2013). Thus, research has examined the posterior parietal cortex due to its hypothesized role in attending to memory representations. It has been suggested that regions of parietal cortex are critical for episodic retrieval because of their role in either the formation of memory representations or shifts in attention for decision making processes, or both (Dobbins et al., 2003).

For example, Manenti et al. (2010a) implemented an rTMS-fMRI design to investigate the causal functioning of both the parietal cortex and DLPFC during encoding and retrieval. The authors applied online 10 Hz rTMS to either the left or right DLPFC or individualized regions of the left or right parietal cortex (either supramarginal or angular gyrus) during either encoding or retrieval. The authors demonstrated word recognition decrements (i.e., slower reaction times) following rTMS to the left parietal cortex (compared to sham). Furthermore, those with larger rTMS-related memory decrements were those who demonstrated a larger degree of right parietal activation during retrieval in the initial fMRI-localization task (Manenti et al., 2010a). One implication from these findings is that different aspects of episodic memory performance (e.g., accuracy, reaction times, subjective confidence) may be supported differentially by the PFC vs. PC.

Additionally, the null memory results reported (Manenti et al., 2010a) contrast with previously reported findings of Innocenti et al. (2010), despite the use of many of the same parameters (i.e., 10 Hz, left and right DLPFC, encoding, and retrieval of words). One possible explanation may be attributable to differences in other parameters, such as stimulation intensity (i.e., 100 vs. 90%). Similarly, the findings of Manenti et al. (2010a) also differed from Köhler et al. (2004) despite implementing many of the same parameters (i.e., online stimulation at the same intensity to the PFC and PC during word encoding). However, differences in the stimulation frequency (i.e., 7 vs. 10 Hz) might account for the different results.

It should be noted that studies often select a stimulation site to have a selective (or, more likely, stronger) effect on one cognitive domain, but it must be acknowledged that stimulated brain regions may be implicated in numerous related cognitive processes. For example, a meta-analysis revealed that rTMS to the DLPFC improved working memory performance (e.g., accuracy, reaction times; Brunoni and Vanderhasselt, 2014). The studies included in the Brunoni and Vanderhasselt (2014) meta-analysis targeted the DLPFC because of its role in short-term (STM)/working memory (WM). Thus, subsequent assessments of the rTMS effects on long-term episodic memory should be interpreted based on possible modulation of STM/WM processes on encoding and maintenance processes that may subsequently result in modulation of long-term memory. Indeed, a number of studies have examined the effects of rTMS to the DLPFC during STM/WM and has assessed the effects of rTMS on subsequent long-term memory (Nilakantan et al., 2017; Marin et al., 2018). For example, Marin et al. (2018) found that continuous theta burst (cTBS) to the right DLPFC prior to the encoding of objects and their spatial location was impaired for subsequent long-term memory tasks (i.e., object recognition and spatial location recall), but short-term memory performance (i.e., short-term spatial location recall) was left unaffected compared to controls.

Taken together, the findings reviewed thus far build upon previous correlational neuroimaging findings that the PFC, PC, and MTL are important for episodic memory, but systematic examination of encoding, consolidation, and retrieval processes, as well as potential interactions among neocortical regions, is needed. For example, the functional roles of these regions in recollection and familiarity (Yonelinas, 2005) remains to be fully understood.

### The Role of Stimulation Frequency, Intensity, and Timing

How rTMS can enhance or impair memory (or leave it unaffected) depends on numerous factors regarding the ways in which the rTMS protocols are implemented. A detailed review of all technical considerations regarding rTMS is beyond the scope of the current paper (for details, see Kammer et al., 2001; Rossi et al., 2009; Sauvé and Crowther, 2014). The main stimulation characteristics considered here are stimulation frequency, intensity, and timing. A common approach in selecting a stimulation frequency generally involves applying a protocol hypothesized to either enhance cortical excitability, such as a high frequency (>5 Hz) or intermittent theta burst (iTBS) protocol, or suppress cortical excitability, such as a low frequency (<1 Hz) or cTBS protocol (Huang et al., 2005; Hallett, 2007). However, these hypotheses stem largely from physiological findings that have been investigated in the motor cortex with the assumption that this remains true for other cortical regions.

Another way in which different stimulation frequencies may modulate neural activity is through altering neural oscillatory activity through the resetting of natural oscillations, and in some cases, driving neural entrainment that may influence communication between brain regions and cognitive performance (Luber and Lisanby, 2014). Importantly, this modulation of neural activity is assumed to be specific to functionally relevant frequencies. Because relevant oscillations may vary in a continuous fashion between tasks, across phases of a task, and/or between individuals, considerable research is needed to fully understand how rTMS frequency can modulate episodic memory processes. A major aim of the current meta-analysis is to help elucidate the aggregate and complex interactions associated with the effects of different rTMS frequencies on episodic memory.

One of the many factors that will influence the effectiveness of rTMS is stimulation intensity. Generally, researchers will adjust stimulation intensity based upon an individual's resting or active motor threshold (RMT, AMT; Ngomo et al., 2012), which is defined as the minimal stimulator intensity needed to induce a muscle evoked potential in 5 out of 10 occurrences in either a relaxed (RMT) or actively engaged muscle (AMT). Prior work has found evidence that low (below) and high (above) stimulation intensities mirror facilitatory vs. inhibitory neural activity that is also associated with enhancements vs. decrements in behavioral outcomes (Silvanto et al., 2018). In addition, recent findings have shown that the effects of stimulation intensity on cortical excitability also may interact with specific frequencies, which in turn can lead to divergent effects on cognitive performance (Chung et al., 2018). Furthermore, the effects of varying stimulation intensity may covary with numerous factors such as the “brain state” or the cortical excitability of targeted and functionally coupled regions at the time of stimulation (Romei et al., 2016). Thus, a systematic investigation of the effects of stimulation intensity and how it interacts with frequency is needed. Elucidating the effects of varying stimulation intensity on episodic memory was another aim of the current study.

When stimulation is applied (i.e., stimulation timing parameter) is also an important factor that enables researchers to investigate the time course of episodic memory processes. The timing of the stimulation protocol reflects differences in whether stimulation occurs simultaneously with a cognitive process (online; e.g., rTMS during picture encoding) or prior to or following a cognitive process (offline; e.g., rTMS prior to picture encoding). One reason that online and offline stimulation may affect episodic memory differently is that they may affect different mechanisms, with online stimulation having an immediate influence on brain activity while offline stimulation often results in after-effects up to an hour after stimulation (Bergmann et al., 2016). In other words, online approaches can interfere or enhance neural activity through immediate depolarization of targeted neurons, while offline approaches can modulate long-term potentiation- or long-term depression-like plasticity mechanisms to either enhance or inhibit episodic memory processes. In a previous meta-analysis of different forms of non-invasive brain stimulation on various cognitive measures, differences in stimulation timing were found, with larger enhancing effects occurring for online stimulation (Hsu et al., 2015). Importantly, stimulation timing may interact with stimulation intensity, which could lead to facilitating or impairing effects on cognition (Silvanto and Cattaneo, 2017).

### The Importance of Proper Control Conditions

The implementation of a proper control conditions is important to note given the variety of different control comparisons that are available. A proper control comparison is critical in order to assess whether the observed effects on performance are due specifically to the effects of applying TMS to the targeted region as opposed to some other variable, such as non-specific effects associated with the sensations of stimulation (Sandrini et al., 2011). Active stimulation control conditions that involve stimulating some other brain region that is not hypothesized to be as involved in the specific process of interest as the experimentally targeted region at the same intensity and frequency in both conditions are ideal because they most closely replicate the same aspects of the experimental/treatment TMS procedures. Sham stimulation controls (e.g., sham coil or tilting the coil away from the scalp) are implemented to mimic the non-specific effects of TMS (i.e., the discharging sound and scalp contact of the coil). “No TMS” conditions provide the least amount of control for non-specific effects of rTMS. In order to test if the type of control condition moderated the size of rTMS effects on episodic memory we examined whether there were systematic differences between effect sizes associated with comparisons to active, active vertex, sham, or no TMS control conditions.

## The Present Study

A clear understanding of when episodic memory is enhanced, impaired, or left unaffected should depend on a wide variety of factors regarding the ways in which the rTMS protocols are implemented, and a subset of these factors can be roughly classified into specific rTMS parameters (e.g., frequency, intensity, timing, stimulation site, and control comparisons) and experimental design characteristics (e.g., memory processes: encoding vs. consolidation vs. retrieval, and retrieval type: familiarity vs. recollection). Contrasting the effects of different parameters allows researchers to test hypotheses about the role of specific neurocognitive processes that support different aspects of episodic memory. For example, rTMS could be used to influence many different processes associated with the acquisition of an event (encoding), its storage and preservation over time (consolidation processes), and/or the ability to remember the event (retrieval processes). Collectively, these different stages may engage both, similar, or distinct brain regions and, thus, the targeted brain regions and the nature of stimulation should result in variable effects. With this in mind, we conducted our survey of the literature to examine how rTMS has been shown to modulate episodic memory with a specific focus on how, when, and where rTMS was applied, as well as how episodic memory was measured.

A significant hurdle with understanding how rTMS impacts episodic memory is that most studies differed in a number of ways, such as in stimulation parameters (e.g., frequency, intensity, timing, and stimulation site), or with respect to how episodic memory was measured. Such differences make it difficult to pinpoint the specific protocols that will enhance or impair episodic memory. In the current study, we conducted a comprehensive systematic review of the literature with the aim of identifying the aggregate effects of rTMS on episodic memory and interactions among various factors. The focus of the meta-analysis was on healthy younger adults due to the small number of older adult rTMS studies. To the extent of our knowledge, this is the first meta-analysis to investigate this relationship. Such a meta-analysis is important for drawing consistent conclusions regarding the circumstances that lead to rTMS impacting episodic memory.

In order to carry this out, our primary aim was to investigate the effects of potential moderators of effect sizes associated with specific rTMS parameters or experimental design characteristics. Specific rTMS parameters included: stimulation frequency (1, 5, 10, 20, iTBS, cTBS), intensity (above, at, and below 100% MT), timing (online vs. offline), targeted hemisphere (left vs. right), targeted cortical region (frontal cortex vs. parietal cortex), and control conditions (active, active vertex, sham, and no TMS). Experimental design moderators included: memory processes stimulated (encoding vs. retrieval) and retrieval type (recollection vs. familiarity). Relatedly, we attempted to elucidate the complex relationship between rTMS and episodic memory by examining two-way interactions between factors that are hypothesized to interact. This allowed us to begin to bridge the gap in the understanding of the circumstances in which rTMS will lead to memory enhancement, impairment, or leave memory unaffected. Although it is not yet possible to conduct a similar meta-analysis of the effects of rTMS on episodic memory in older adults and some clinical conditions, we provide a brief narrative review of the available studies for comparison.

## Method

We pursued all possible pertinent articles that reported data (e.g., group means, mean differences, tables, and bar graphs with error information) regarding the relationship between rTMS and episodic memory performance, and coded relevant parameters for potential moderator variables. First, we detail how the literature search was conducted for the systematic review, including the inclusion criteria that were implemented and selection protocol. Next, we discuss the subset used for the meta-analysis, which will focus on rTMS and episodic memory in healthy younger adults given that they account for a majority of the findings reported in the literature. Details for the coding system and how effect sizes were calculated and implemented in a three-level random effects meta-analysis are reported below.

### Literature Search

**Figure 1:**
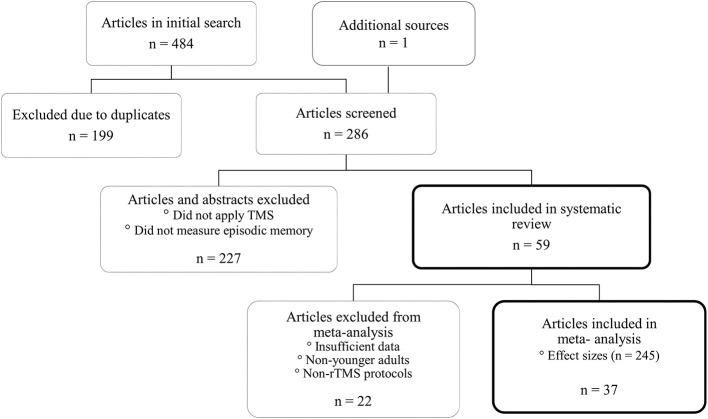
A total of 59 articles were included in the systematic review. The meta-analysis focused on healthy younger adults and rTMS in episodic memory resulting in 37 articles with 245 effect sizes.

An exhaustive PubMed search was conducted to select studies for the review and meta-analysis with a combination of the following search terms: “transcranial magnetic stimulation,” “TMS,” “rTMS,” “Theta burst stimulation,” “TBS,” “memory,” “episodic memory,” “long term memory,” “associative memory,” “recollection,” “mild cognitive impairment.” The search for studies ended in July 2018. Our literature search yielded 484 studies from PubMed, and references from these relevant articles were examined for possible inclusion in our analysis. In line with previous protocols, when the title and abstract were insufficient to identify the study for inclusion, the full-text underwent further examination. In cases of uncertainty, articles were examined by the authors and research assistants and removed based on mutual agreement. The most frequent reasons for exclusion were that articles (a) did not apply TMS or (b) did not assess episodic memory, see Figure 1.

### Inclusion Criteria and Selected Studies

For inclusion in the systematic review, the studies were limited to (1) single pulse TMS, paired pulse TMS, rTMS, or TBS protocols that measured (2) episodic memory with a free-recall, cued-recall, or recognition test (3) in healthy younger adults, (4) healthy older adults, or (5) older adults with a clinical condition, (6) and those that were published in English.

A total of 59 studies were incorporated into the systematic review based on the search terms and inclusion criteria. However, inclusion in the meta-analysis was focused on rTMS studies of healthy younger adults. Seven of the remaining studies were excluded from the meta-analysis because there was insufficient reporting of data necessary to calculate effect sizes (e.g., means, SDs, t-, or F-values). Thus, 37 articles examining the effects of rTMS on episodic memory in healthy younger adults were included in the meta-analysis. See Figure 1 for a flow diagram illustrating this process. A total number of 245 effect sizes were extracted from the selected studies. For experiments that pooled participants' data across multiple studies, only participants who were uniquely recruited for each study were analyzed. Details about the calculation of effect sizes are described below in the data extraction section.

### Quality Assessment

In order to assess the methodological quality of the selected studies, we were guided by the assessment criteria recommended by Higgins et al. (2008), which is to categorize study characteristics as low risk, unknown risk, or high risk based on whether there was random allocation of participants to conditions, blinding of participants, blinding of experimenter, blinding of experimenter assessing the outcome measure(s), selective reporting of outcome measures, and participant attrition. Characteristics were coded as unknown when insufficient details were provided; studies were categorized as high risk when it was clear that the conditions were not met, such as when it was implausible for the participants and/or experimenters to have been blind to the administration of the experimental vs. control conditions. We also extended these criteria to include additional assessments pertinent to TMS studies, namely the targeting procedure and type of control condition. For targeting procedure, if a study used structural or functional MRI neuronavigation, international 10–20 EEG positioning system, or distance measurements, they were classified as low risk, unknown risk, and high risk, respectively. A variety of control conditions were implemented, such as active, sham, and no TMS conditions, that were classified, as low, unknown, and high risk, respectively.

### Coding of the Studies

Moderators that were hypothesized to modulate the size of effects of rTMS on episodic memory were coded for each study. In order to code for moderators, the full-text articles were examined for explicitly stated information regarding the moderators of interest. In situations where it was not clearly stated, the moderators were inferred from the study protocol (e.g., the study did not state if stimulation occurred “online” or “offline,” but, if the task figures showed that stimulation occurred prior to a cognitive task, then we coded stimulation timing as offline). Studies were independently coded by both the first author and research assistants to establish reliable coding and any discrepancies were resolved by the authors.

#### Coding of rTMS Parameter Moderators

Frequency was coded as a categorical variable with 1, 5, 10, and 20 Hz being coded as “one,” “five,” “ten,” and “twenty,” respectively. Theta burst frequencies were separated and coded as intermittent theta burst (iTBS) or continuous theta burst (cTBS). One study used a modified iTBS protocol with short iTBS (2 s) trains interleaved with stimulus presentation and was classified as online iTBS. None of the obtained studies implemented an intermediate theta burst (imTBS) procedure. A remaining 15 effect sizes across 3 studies implemented stimulation frequencies (i.e., 6.8, 7, 10.7, 17.5, and 18.7 Hz) that did not fall in these categories and, thus, were not used in analyses examining the effects of frequency.

Motor threshold intensity (MTI) can be calculated numerous ways, such as based upon individual RMT or AMT. Although procedures that estimate motor thresholds using AMT vs. RMT or MEP vs. visually-evoked responses result in slightly lower levels of stimulator intensity (Rossini et al., 2015), there were not enough studies that clearly implemented either RMT or AMT with either MEP or visually-determined thresholds to allow for systematic investigation of the role of different thresholding procedures. Therefore, MTI was treated as a categorical variable. Studies that reported using stimulation parameters at, above, or below 100% of motor threshold were coded as “at,” “above,” or “below,” respectively.

Studies were coded as “online” protocols when stimulation occurred simultaneously with the study's experimental task, such as when rTMS co-occurred with stimulus (e.g., words or pictures) presentation during either encoding or retrieval. Although studies differed with regards to the precise timing of the delivery of TMS pulses, for example, at stimulus onset or varying intervals from stimulus onset, there were not enough studies to examine possible differences in the effects of timing differences in the administration of online rTMS protocols at this time. Therefore, we collapsed across different online timing protocols. Studies where stimulation occurred in isolation without any co-occurring task, such as before or after an encoding or retrieval task, were coded as “offline” protocols.

The hemisphere that was stimulated was coded as “left” or “right.” Studies that targeted cortical regions in frontal cortex or parietal cortex were coded as “FC” and “PC,” respectively. There were too few studies that targeted regions beyond the FC and PC (e.g., occipital cortex) for valid analysis and inference of aggregated effect sizes at this time. Therefore, a meta-analysis investigating the effects of stimulation that targeted either consolidation processes or regions outside the PC and FC was not possible at this time. Again, these gaps in the literature highlight areas where more research is needed.

Lastly, we coded studies based on the type of control condition used for comparison to the primary “treatment” condition of interest in order to assess the potential differences between different types of control conditions. Classification of an “active” control occurred when experimental stimulation was compared to any active rTMS control site except for the vertex. A “vertex” control comparison was coded separately. When studies implemented either a sham coil or applied sham stimulation by tilting the coil away from the participant's head, they were coded as “sham” controls. In situations where an experimental stimulation group was compared to a condition in which no rTMS was applied, they were coded as “no TMS.”

#### Coding of Experimental Design Moderators

The “stimulated memory processes” (encoding or retrieval) were coded as the main memory processes that were most likely to be primarily affected by stimulation based on when stimulation was applied. A study was coded as targeting “encoding” if the stimulation parameters occurred within seconds to minutes prior to, during, or immediately after (e.g., within 400 ms of stimulus offset) encoding each item. For example, conditions with a train of cTBS administered within minutes prior to encoding all items was coded as primarily targeting “encoding.” Similarly, conditions with brief trains of 1 Hz rTMS during picture presentation were coded as targeting “encoding.” Situations in which stimulation occurred shortly after picture presentation offset (i.e., within milliseconds), were coded as targeting encoding. While such protocols may affect both encoding and early consolidation processes, studies were categorized using this range in order to maximize the number of effect sizes for reliable analysis. The critical distinction for this analysis was to compare the aggregate effect size of rTMS protocols that clearly impacted retrieval processes vs. encoding/early-consolidation processes. Unfortunately, there was only one study that applied stimulation during consolidation, which prevented its inclusion in the meta-analysis. If stimulation occurred immediately before or during a retrieval task (e.g., cTBS prior to attempting to retrieve all previously encoded words or 1 Hz rTMS during presentation of each picture in a recognition test), the study was coded as primarily targeting “retrieval.”

To test for a difference in the effects of rTMS on episodic retrieval processes that are hypothesized to be distinct by dual-process theories, effect sizes were extracted for conditions hypothesized to primarily measure either recollection or familiarity. Conditions were aggregated and coded as assessing “recollection” if they assessed free recall, cued recall, “remember” responses in remember/know recognition memory paradigms, and paradigms that reported associative- or source-recognition memory performance. Associative- or source-recognition estimates (i.e., associative- or source-hits, corrected recognition scores, or d′) were extracted from recognition memory tasks that required the retrieval of qualitative information about the study of a specific piece of information (e.g., if participants studied a soccer ball object and were asked to recall its spatial location), as in source memory paradigms. Conversely, conditions were aggregated and coded as assessing “familiarity” if they assessed and reported recognition memory performance (e.g., item memory hit rates, corrected scores, d′) on tests that did not require additional qualitative information to be retrieved. This coding scheme included “know” responses for remember/know paradigms.

### Data Extraction

Means (M), standard deviations (SD), and sample sizes (*n*) were obtained for relevant episodic memory outcome measures for each study. When standard errors (SE) were reported, they were converted to SD. In cases where there was insufficient statistical information provided in the text or tables, they were collected from graphical representations using GetData Graph Digitizer data extraction software (downloaded at http://www.getdata-graph-digitizer.com). In the event that insufficient data could be obtained, attempts were made to contact the corresponding authors for additional data. Descriptive statistics and effect size calculations were done independently by the first author and research assistants to cross-check the calculations and resolve any discrepancies. The table of experiments included in the meta-analysis, their moderator variables, and all of the effect sizes that were extracted from the experiments are presented in an open-access database here: https://osf.io/6kbqe/?view_only=226e600d6e1546748b52f6bbfc593140. For additional information or to contribute additional data, please contact the first author. Our intention for sharing the data is so that effect size calculations can be confirmed and so that additional studies can be continuously added as the literature grows, which will facilitate future meta-analysis and systematic investigation.

Effect sizes for between-subject comparisons were computed with the following formula:

$$\begin{array}{l} d=\frac{\left(M_{Experimental}-\;M_{Control}\right)}{SD_{Pooled}} \end{array}$$

When studies reported comparisons between gain scores (e.g., Post-experimental—Pre-experimental) the effect size was calculated with the following formula:

$$\begin{array}{l} d\;=\frac{\left(\left(M_{Experimental,\;Post}-\;M_{Experimental,\;Pre}\right)-\left(M_{Control,\;Post}-\;M_{Control,\;Pre}\right)\right)}{SD_{Pooled,\;Post}} \end{array}$$

Effect sizes for within-subject comparisons were calculated with the following formula:

$$\begin{array}{l} d=\;\frac{\left(M_{Experimental}-\;M_{Control}\right)}{\left(\frac{\left(SD_{Experimental\;}+\;SD_{Control}\right)}{2}\right)} \end{array}$$

This was done to obtain an accurate effect size estimate for within-subject designs (Cumming, 2012) using the commonly recommended approach for meta-analyses (Lakens, 2013). Alternative routes that address dependencies in within-subject designs, such as averaging effect sizes, can lead to a reduction in statistical power and the loss of information can result in less precise estimates and *SE*'s (Hedges and Pigott, 2001; Cheung, 2014). In a similar vein, limitations also arise when calculating Cohen's *d*_rm_, which attempts to account for the dependencies by correcting for the correlation in within-subject designs. As correlations between conditions are rarely reported, estimations may be too conservative, especially when correlations are high (Lakens, 2013).

Hedge's *g* and *g*_av_ effect sizes were calculated, which provides a less biased estimate of the true effect than Cohen's *d*, especially for studies with small sample sizes (Hedges and Olkin, 1985). In order to do this, we first calculated Cohen's *d* and then applied a Hedge's *g* correction (see Cumming, 2012) with the following formula:

$$\begin{array}{l} g=d\;\times \;\left(1-\frac{3}{\left(4\left(n_{Experimental}+\;n_{Control}\right)-9\right)}\right) \end{array}$$

With the exception of 5 studies, we were able to collect multiple effect sizes per study. Thus, we can no longer assume statistical independence, as within-study effect sizes may be more similar than effect sizes from other studies. Thus, dependent effect sizes may contain redundant information that becomes generally less informative when effect sizes are correlated. This form of dependency may occur when multiple effect sizes can be calculated from multiple outcome measures (Van den Noortgate et al., 2013). For example, in examining the effects of rTMS on episodic memory, a study can test a set of stimulation parameters (e.g., 10 Hz rTMS at 100% AMT to the DLPFC) on multiple memory outcomes, such as recall, recognition and source memory. A second dependency occurs when effect sizes are correlated in situations where an experimental condition is compared to multiple control groups or multiple experimental conditions are compared to one control group. For instance, many of the studies compared experimental stimulation to combinations of active control, sham, or no stimulation conditions, while other effect sizes were comparisons between multiple experimental stimulation conditions to a single control condition. This can be problematic in meta-analyses and increase type 1 errors if dependent effect sizes are assumed to contain unique information. Therefore, to address multiple dependency concerns we conducted a three-level random effects meta-analysis with Hedge's *g* and *g*_av_ corrections, which is detailed below (for additional information on dependencies and three-level models see, Van den Noortgate et al., 2013).

### Three Level Modeling

We conducted a three-level random effects model using the Metafor package in R (Viechtbauer, 2010). The typical random effects approach allows for effect sizes to vary due to sampling variation and due to differences between studies. In order to account for studies that contain multiple effect sizes, we can extend this model to a three-level random effects model to take into account the correlation of within-studies effect sizes. This approach does not assume independence among effect sizes as is done with other meta-analysis techniques. Multilevel modeling allows for an alternative for dealing with the dependency of effect sizes when correlations between outcomes are not reported or there is insufficient data to estimate the dependency (for additional details, see Assink and Wibbelink, 2016). The hierarchical structure of the data allows effect sizes to vary between participants (level 1; sampling variance), outcomes (level 2; within studies), and studies (level 3; between studies). In this case, individual studies (denoted by the subscript *k*) can contain one or multiple outcomes (denoted by the subscript *j*). The studies (i.e., Study ID) and individual effect sizes (i.e., Effect Size ID) were entered into the model as random effects. The simplest model with no moderators and three residuals (v_k_, u_jk_, e_jk_) that are assumed to be normally distributed with a mean of zero is:

$$\begin{array}{l} {\text{ES}}_{\text{jk}}=\beta_{0}+{\text{v}}_{\text{k}}+{\text{u}}_{\text{jk}}+{\text{e}}_{\text{jk}} \end{array}$$

where an observed effect size for outcome *j* within study *k* is represented by ES_jk._ The overall population effect size mean across all outcomes and studies is represented by β_0._ The v_k_ element is the random mean deviation effect in study *k* from the overall effect. The random deviation of *j*th population effect in study *k* from the mean effect in study *k* is denoted by u_jk._ Lastly, e_jk_ is the random residual error from the population effect due to sampling variation. Parameter estimates in the meta-analysis include the between-study variance ($\sigma_{\text{v}}^{2}$) and within-study variance ($\sigma_{\text{u}}^{2}$), and the sampling variance is not reported because it is calculated from reported data prior to the analysis. Therefore, observed significant between-study variance differences reflect that effect sizes systematically vary between studies to a greater degree than would be expected due to random differences in sampling variance (due to chance). Meanwhile significant within-study variance above zero signifies that the observed effects within a study vary across individuals to a greater degree than chance.

In order to implement a parsimonious model, we examined if our three-level model provided a better model fit over more simplistic models with multiple ANOVAs. There was a significant difference between a single and two-level model (within-study variance), likelihood ratio test (LRT) = 41.75, *p* < 0.01. There was also a significant difference between two and three-level models (between-study variance), LRT = 41.83, *p* < 0.01. Thus, the three-level modeling of the dependent effect sizes provided a better model fit than treating the effect sizes as independent.

### Publication Bias

A prevalent issue in systematic reviews and meta-analyses is how to address missing data due to publication bias. This problem occurs when significant results are more likely to be published than non-significant findings. If left unaccounted for, publication bias may lead to spurious results. In this instance, publication bias may lead to the true effect of rTMS on episodic memory being inflated. We used two methods to assess the degree of possible publication bias in the data: funnel plots and an extension of Egger's regression method (Egger et al., 1997)

#### Funnel Plots

One approach to identify and estimate missing data is to examine funnels plots for asymmetry (Sterne et al., 2011). In order to do this, a scatter plot is created with the effect size on the x-axis and the sampling variance (or some variation taking into account the sample size) on the y-axis. Publication bias is less likely to be a concern when effect sizes are distributed symmetrically around the mean effect and are clustered around the mean effect size. Conversely, support for possible publication bias manifests visually in these plots with an asymmetrical distribution of effect sizes around the mean effect size, often with larger sampling variances producing larger effect sizes.

Contour-enhanced plots offer an extension of assessing publication bias by drawing a reference line at zero with contour color changes at different significance levels (0.01 and 0.05) as the effect sizes are plotted against precision (1/SE, inverse of the standard error). In this case, publication bias can be examined with missing effect sizes observed in non-significant regions of the plot. However, there are other possible causes of asymmetry besides publication bias. Asymmetry due to heterogeneity of potential moderators is another factor that can influence asymmetry and can be assessed with residual plots. Here, residuals are plotted against their standard errors at different levels of the moderators (e.g., online vs. offline protocols). Similar to the contour-enhanced plots, significant levels can be added, and asymmetry of the moderators can be examined in relation to the predicted effect sizes. In an attempt to address both publication bias and other potential forms of publication bias we examined contour enhanced and residuals plots.

#### Extension of Egger's Test

Additional traditional approaches for formally testing for publication bias, such as trim and fill and Egger's test, are complicated with three-level modeling. Although correcting (e.g., trim and fill) for publication bias is important (Copas, 1999), obtaining more precise estimates through correction methods may only occur if the bias is large (Hedges and Vevea, 1996). Therefore, we decided to implement an extension of Egger's test for more complex models by entering the standard error into the model as a moderator. Essentially, this provides an extension of Egger's regression method which involves estimating funnel plot asymmetry by measuring how much the regression line (effect size vs. precision, 1/SE) deviates from zero. However, future research is needed to determine how to properly assess missing data in three level meta-analyses, as the numerous available methods to measure and correct for publication bias each come with limitations and, to our knowledge, have not been evaluated in the three-level meta-analysis approach.

## Results

The systematic review incorporated 1,532 participants across 59 articles, see Figure 1. This resulted in an average age of 33.12 (18–80) with 61% females and 39% males in the studies. Of the 59 articles, 44 contained rTMS procedures focusing on younger adults, while 12 articles included healthy older adults and those with memory impairments (i.e., aMCI, Alzheimer's disease) or other disorders (i.e., depression, fibromyalgia, alcohol dependency). The small number of studies focusing on older adults made a meta-analysis unfeasible for comparison with younger adults. Therefore, our approach was 2-fold. First, we conducted a three-level meta-analysis focusing on the effects of rTMS on episodic memory in younger adults. Second, we synthesized the results of studies using rTMS to modulate episodic memory in older adults in the form of a narrative review.

A total of 1,016 participants were included in the meta-analysis of the 37 articles that were identified. The average age of participants across the studies was 25.70 years old with 39% male and 61% female. The average sample size was 24.19 (range 10–69). For a breakdown of the study designs and protocols, see Table 1.

**Table 1:** Parameters and results of experiments that applied TMS and assessed episodic memory in healthy younger adults, healthy older adults, and older adults with memory impairment.

**References**	***N***	**TMS**	**Online/Offline**	**Stimulated cognitive process**	**Targeting procedure**	**Target area**	**Control stimulation**	**Outcomes**	**Result**
**YOUNG ADULTS**									
Kahn, 2005	14 (YA)	Single-pulse fixed at 70% of stimulator output	Offline post stimulus onset (none, 250, 300, 320, 340, 350, 360, 370, 380, 390, 400, & 600 ms) Within subjects	Encoding of words	sMRI and fMRI	Left and right VLPFC	No TMS	Recognition and confidence ratings of words	↓ confidence ratings during left VLPFC stimulation with greatest decrements at 380 ms stimulation compared to baseline. ↑ in confidence ratings for familiar words with a peak at 380 ms stimulation to the right VLPFC compared to no TMS.
Machizawa et al., 2010	15 (YA)	Paired pulse at 120% MT	Online at stimulus offset. First pulse at 350, 750, or 1,150 ms. Second pulse 40 ms after	Encoding of words	sMRI	Left and right inferior frontal gyrus	Active vertex and no TMS	Recognition of words	↓ in recognition performance following left and right IFG stimulation collapsed across delays compared to control.
Gagnon et al., 2010	18 (YA)	Paired pulse 0.5 Hz with an ISI of 3 ms at 90% MT	Online 400 ms after stimulus presentation. Within subjects	Encoding or retrieval of verbal or non-verbal material	10–20 system	Right PFC and left DLPFC during encoding, and right DLPFC during retrieval	Sham with coil angled away	Recognition of words or shapes	Encoding: ↓ in discrimination rate (hits-false alarms) following left DLPFC stimulation compared to sham. Retrieval: ↓ in hit and discrimination rates following right DLPFC compared to left DLPFC stimulation.
Epstein et al., 2002	15 (YA)	6 paired pulses/trial with 60 ms ISI at 120% MT	Offline following word and picture encoding; within subjects for left, right, and active between subjects for no TMS	Encoding of pictographs and unfamiliar patterns	Distance measurements	Right DLPFC and left DLPFC	Active vertex and no TMS	Associative recall	↓ in associative memory after right DLPFC compared to left DLPFC, sham, and no TMS.
^*^Turriziani et al., 2012 Exp 1	40 (YA)	1 Hz rTMS at 90% MT	Offline with 2 sessions separated by 6 h during 10 min delay between encoding and recognition Within subjects (real vs. sham TMS) and Between subjects (left vs. right DLFPC)	Retrieval of faces or buildings	10–20 system	Right DLPFC (i.e., F4) or left DLPFC (i.e., F3)	Sham with coil angled away	Recognition of faces or buildings	↑ recognition with 1 Hz rTMS to right DLPFC compared to sham.
^*^Turriziani et al., 2012 Exp 2	40 (YA)	1 Hz rTMS at 90% MT	Offline with 2 sessions separated by 6 h during 10 min delay between encoding and recognition Within subjects (real vs. sham TMS) and Between subjects (left vs. right DLFPC)	Retrieval of words	10–20 system	Right DLPFC (i.e., F4) or left DLPFC (i.e., F3)	Sham with coil angled away	Recognition words	↑ in recognition with 1 Hz rTMS to right DLPFC compared to sham.
^*^Turriziani et al., 2012 Exp 3.	20 (YA)	iTBS for 192 s at 80% AMT	Offline with 2 sessions separated by 6 h during 10 min delay between encoding and recognition Within subjects (real vs. sham TMS) and Between subjects (left vs. right DLFPC)	Retrieval of faces or buildings	10–20 system	Right DLPFC (i.e., F4) or left DLPFC (i.e., F3)	Sham with coil angled away	Recognition of faces, buildings, or words	↓ in recognition after iTBS to the right DLPFC compared to sham.
^*^Wais et al., 2012	22 (YA)	1 Hz rTMS for 10 min at 100% MT	Offline immediately prior to retrieval Within subjects	Retrieval of scenes and words during visual distraction vs. no distraction	sMRI	Left mVLPFC (MNI: −54, 28, 6)	Active vertex (MNI: 0, −30, 76) and sham with coil angled away	Cued recall of scenes and words	↓ in memory performance in distraction trails compared to no distraction after left VLPFC stimulation.
^*^Sandrini et al., 2013 Exp 1	30 (YA)	1 Hz rTMS for 15 min (900 pulses) at 100% MT	Offline 24 h after encoding and 24 h before retrieval Between subjects (With cue, no cue, or vertex)	Reconsolidation of object word pairs	10–20 system	Right DLPFC with spatial contextual cue (i.e., F4)	Exp 1. Active DLPFC non-spatial contextual cue & active vertex	Free recall of words at 24 h delay	↑ in words recalled at 24 h delay following stimulation compared to active vertex and active non-cue PFC stimulation.
^*^Sandrini et al., 2013 Exp 2	20 (YA)	1 Hz rTMS for 15 min (900 pulses) at 100% MT	Offline Between subjects (With cue or vertex)	Reconsolidation of object word pairs	10–20 system	Right DLPFC with spatial contextual cue (i.e., F4)	Exp 2. Active vertex	Free recall of words at 1 h delay	No differences in recall at 1 h delay following stimulation to active vertex and active cue PFC stimulation
^*^Thakral et al., 2017	16 (YA)	1 Hz rTMS for 10 min at 70% maximum output	Offline (occurs during non-relevant odd/even judgment task prior to episodic memory or simulation task) Within subjects	Episodic simulation, memory, and word association task	sMRI	Left Angular Gyrus (MNI:-48,−64, 30),	Active vertex	Episodic simulation and memory associated with words (30 min delay)	↓ in episodic internal details in episodic simulation and memory following left angular gyrus stimulation compared to control. Increase in external episodic details following left angular gyrus stimulation compared to control. Increase in perceived difficulty of simulation and episodic memory task following left angular gyrus stimulation compared to control.
^*^Ye et al., 2018	18 (YA)	1 Hz rTMS for 20 min (1,200 pulses) at 110% AMT	Offline immediately prior to encoding for 2 sessions on different days Within subjects	Temporal order judgments and confidence ratings	sMRI	Precuneus (i.e., MNI: 6, −70, 44)	Active vertex	Recognition of temporal sequence of images and confidence ratings	↓ in metacognitive efficiency following precuneus stimulation compared to control. No differences in hit rates between conditions.
^*^Rami et al., 2003	16 (YA)	5 Hz and 1 Hz rTMS for 10 s trains at 90% MT	Online at the start and end of episodic memory task Within subjects	Working or logical memory, verbal fluency, and episodic memory task	Distance measurements	Right DLPFC, left DLPFC, and right cerebellar	No TMS	Rivermead test	↓ in logical memory and verbal episodic memory following 5 Hz rTMS to the left DLPFC compared to right DLPFC stimulation and 1 Hz left DLPFC stimulation.
^*^Balconi and Cobelli, 2015	69 (YA)	5 Hz rTMS (90 total trains) at 100% MT	Online during word/picture presentation with 2 sessions (1 for word retrieval and 1 with picture retrieval) Within subjects	Retrieval of emotional words and pictures	10–20 system	Left DLPFC (medial frontal gyrus; Talairach: −10,40,25)	Active (i.e., Pz) and sham with coil angled away	Recognition and reaction times to emotional words and pictures	↑ in memory performance and reduced reaction time for high arousal positive words and pictures following left DLPFC stimulation compared to active control and sham.
^*^Köhler et al., 2004	12 (YA)	7 Hz rTMS at 100% or 60% (n = 2) RMT	Online 200 ms after stimulus onset during encoding and intermixed with no stimulation per condition Within subjects	Encoding of words	sMRI and fMRI	Left IPFC	Active (right IPFC and left parietal cortex)	Recognition of words	↑ in word recognition after left IPFC stimulation compared to both control conditions.
^*^Hawco et al., 2013	35 (YA)	10 Hz rTMS at 100% RMT for 2 s trains (2,880 pulses)	Online during word pair presentation and 30–35 min before retrieval Within subjects	Encoding strategies for word pairs	10–20 system	Left DLPFC (i.e., F3)	Active vertex (i.e., Cz)	Cued word recall	↑ in word pairs recall for self-initiated low strategy users and ↓ recall for self-initiated high strategy users following left DLPFC stimulation compared to control.
^*^Innocenti et al., 2010	18 (YA)	10 Hz rTMS for 500 ms at 90% MT	Online during word presentation Within subjects	Deep or shallow encoding of words	10–20 system	Right DLPFC & left DLPFC	Active vertex and no TMS	Recognition of words	No benefits of deep encoding after left DLPFC stimulation.
Manenti et al., 2010a	11 (YA)	10 Hz (eleven pulses/train) rTMS at 100% RMT	Online during word presentation at encoding and retrieval Within subjects	Encoding or retrieval of abstract and concrete words	sMRI	Right DLPFC (i.e., MNI: 55, 16, 40), left DLPFC (i.e., MNI: −37,26,49), left parietal cortex (i.e., MNI:−35, −56, 43), and right parietal cortex (i.e., MNI: 44, −55, 44)	Sham with a plywood spacer (i.e., Cz)	Recognition of words	Retrieval: ↓ in word retrieval reaction times after right DLPFC and left parietal cortex stimulation for abstract words.
^*^Feurra et al., 2010	12 (YA)	10 Hz rTMS at 110% RMT	Online immediately at face onset Within subjects	Encoding of faces with context or no context	sMRI	Left occipital face area (Talairach: −42, −74,−8) & left inferior frontal gyrus (Talariach: −49,23,13)	Active vertex and no TMS	Recognition of faces	↓ in retrieval after left occipital face area stimulation. ↓ in no context face retrieval following left IFG stimulation.
^*^Koen et al., 2018	20 (YA)	10 Hz (5 pulses) at 100% MT	Online 500 ms post stimulus onset Within subjects	Encoding of word pairs	sMRI and fMRI	Left angular gyrus	Active vertex	Recognition and confidence ratings of the word pairs	↑ confidence for incorrect intact judgments to rearranged pairs and incorrect rearranged judgments for intact pairs. No difference in recollection or familiarity-driven recognition.
^*^Rossi et al., 2011 Exp 1	15 (YA)	20 Hz rTMS at 90% RMT	Online during encoding at 100, 200, 300, 400, and 500 ms stimulus offset Within subjects	Encoding of indoor and outdoor scenes	10–20 system	Left DLPFC	Sham to left DLPFC	Recognition of scenes	↓ in recognition accuracy following left DLPFC stimulation delivered at a delay of 500 ms compared to other active conditions.
^*^Rossi et al., 2011 Exp 2	13 (YA)	20 Hz rTMS at 90% RMT	Online during encoding at 100, 300, and 500 ms stimulus offset Within subjects	Encoding of indoor and outdoor scenes	10–20 system	Right DLPFC	Active vertex	Recognition of scenes	No difference in recognition accuracy across all delays following right DLPFC stimulation.
^*^Galli et al., 2017	24 (YA)	20 Hz rTMS for 500 ms at 90% MT	Online during word presentation or at 0, 100, 200, 300, 400 ms stimulus offset Between subjects (DLPFC vs. VLPFC). Within subjects (word offset, 100, 200, 300, 400 ms stimulation, active and no TMS)	Deep or shallow encoding of words	10–20 system	Left DLPFC (i.e., F3) or left VLPFC (i.e., F7)	Active vertex and no TMS	Recognition of words	Collapsing across levels of processing: ↓ in word accuracy for left VLPFC early post encoding stimulation compared to late post encoding stimulation. No differences online or late post encoding with left VLPFC stimulation. No differences for DLPFC stimulation.
^*^Turriziani et al., 2008	16 (YA)	20 Hz rTMS	Online from 0 to 300 ms and from 300 to 600 ms after stimulus onset Within subjects	Encoding and retrieval faces	10–20 system	Left DLPFC (i.e., F3), & right DLPFC (i.e., F4)	Sham with coil angled away	Remember/Know judgments to visual stimuli	↓ in recollection performance following right DLPFC stimulation compared to left DLPFC stimulation during encoding. ↓ in familiarity performance following left and right DLPFC stimulation during encoding.
^*^Rossi et al., 2001	13 (YA)	20 Hz rTMS at 90% RMT	Online at picture presentation during encoding or retrieval Within subjects	Encoding or retrieval of pictures	10–20 system	Right DLPFC (i.e., F4) and left DLPFC (i.e., F3)	Sham with coil tilted 90 degrees and no TMS	Recognition of pictures	Encoding: ↓ picture retrieval after left compared to right DLPFC stimulation. Retrieval: ↓ picture retrieval after right DLPFC compared to left stimulation.
Sandrini et al., 2003	12 (YA)	20 Hz rTMS for 500 ms at 90% RMT	Online during word presentation Within subjects	Encoding or retrieval of word pairs	10–20 system	Right DLPFC (i.e., F4) and left DLPFC (i.e., F3)	Sham with coil tilted 90 degrees and no TMS	Recognition of word-pairs	Encoding: ↓ word-pair retrieval during left and right DLPFC stimulation for unrelated word pairs compared to related words. Retrieval: ↓ word pair retrieval during right DLPFC stimulation for unrelated word pairs compared to related words.
^*^Martin-Trias et al., 2018	68 (YA)	20 Hz rTMS at 90% RMT	Online 900 ms trains 500 ms after stimulus onset for 3 sessions (i.e., day 1, 2, 3) Within subjects	Encoding of pictures	sMRI and fMRI	Left DLPFC (MNI: −42, 10,30)	Active vertex (i.e., Cz)	Recognition of pictures	↓ hit rates for pictures following left DLPFC stimulation compared to active control for day 2. No memory differences following left DLPFC stimulation compared to active control for day 3.
^*^Floel et al., 2004	15 (YA)	20 Hz rTMS for 500 ms at 90% MT	Online during encoding word or picture presentation for 2 sessions Within subjects	Encoding of words and abstract shapes	sMRI and 10–20 system	Right PFC (i.e., BA 45) and left PFC (i.e., BA 47)	Sham with coil angled away and no TMS	Retrieval (remember, familiar, new judgments) of words and abstract shapes	↓ verbal material after left PFC stimulation compared to right PFC and sham conditions. ↓ in nonverbal material after right PFC stimulation compared to left PFC and sham conditions.
^*^Rossi et al., 2004	37 (YA)	20 Hz rTMS at 90% MT	Online during picture presentation for 500 ms Within subjects	Encoding or retrieval of pictures	10–20 system	Right DLPFC (i.e., F4) or left DLPFC (i.e., F3)	Sham with coil angled away and no TMS	Recognition of pictures	Encoding: ↓ picture retrieval, left more than right DLPFC. Retrieval: ↓ picture retrieval, right more than left DLPFC.
^*^Rossi et al., 2006	42 (YA)	20 Hz rTMS for 500 ms at 90% or 120% RMT	Online during picture presentation Between subjects (active vs. sham) Within subjects (left and right PC and no stimulation)	Encoding or retrieval of pictures	10–20 system	Left parietal cortex (i.e., P3) and right parietal cortex (i.e., P4)	Sham with coil angled away and no TMS	Recognition of pictures	Encoding: ↓ picture retrieval after left compared to right DLPFC stimulation Retrieval: ↓ picture retrieval after right DLPFC compared to left stimulation. No difference between PC conditions.
^*^Sestieri et al., 2013	14 (YA)	20 Hz rTMS for 150 ms at 100% MT	Online during retrieval at picture onset for 2 sessions done on consecutive days. Encoding was session 1 and retrieval session 2 Within subjects	Retrieval of pictures	sMRI and fMRI	Left angular gyrus (i.e., Talairach: −42, −68, 27), left superior parietal lobe (Talariach: −23, −58, 49)	Sham with coil angled away	Recognition of pictures, source memory and confidence ratings	↓ in picture recognition following stimulation of left angular gyrus compared to SPL, but not to sham. ↓ recollection details by altering response bias compared to both SPL and sham conditions.
^*^Manenti et al., 2010b	14 (YA)	10 Hz rTMS at 90% MT	Online 100 ms after trial onset during retrieval for 700 ms Within subjects (right DLPFC vs. left DLPFC vs. sham) Between subjects (strategy users vs. non-users)	Retrieval of face-name associations	Estimated Talairach	Right DLPFC (i.e., Talairach: 35,24,48) and left DLPFC (i.e., Talarach: −35, 24, 48)	Sham with coil angled away	Retrieval of face-name associations	↓ in association retrieval after right DLPFC in strategy users compared to left and sham conditions. ↓ in association retrieval after left DLPFC in no-strategy users compared to right and sham conditions.
^*^Marin et al., 2018	28 (YA)	cTBS for 40 s (600 pulses) at 80% or 20% MT	Offline prior to encoding with 2 sessions separated by ~48 h. Session 1 no TMS. Session 2 DLPFC or Sham Between subjects (DLPFC vs. Sham)	Active and passive retrieval of object locations	sMRI	Right DLPFC (i.e., MNI: 28, −1, 68)	Sham at 20% MT	Cued object recall and spatial recall	↓ in object and spatial recall for active retrieval following stimulation compared to passive retrieval stimulation and sham conditions.
^*^Blumenfeld et al., 2014	26 (YA)	cTBS for 30 s (450 pulses) at 80% AMT	Offline immediately before encoding and 50 min before retrieval Within Subjects (PFC vs. vertex) Between subjects (DLPFC vs. VLPFC)	Encoding of words	sMRI and fMRI	DLPFC (i.e., MNI: −53, 38, 12) or VLPFC (i.e., MNI: −43,35, 30)	Active vertex	Recognition of words	↑ in word recognition for DLPFC stimulation compared to control. ↓ in recognition for VLPFC compared to control.
^*^Berkers et al., 2017	58 (YA)	cTBS for 40 s at 80% AMT	Offline immediately prior to encoding with recall occurring immediately after Between subjects	Encoding of auditory associated words (DRM-paradigm)	10–20 system	Medial prefrontal cortex	Active vertex (i.e., Cz) and no TMS	Free recall and recognition of words	↓ in false recall of critical lures following cTBS to mPFC compared to active control and no stimulation. No difference in veridical recall or recognition across conditions.
^*^Ryals et al., 2016	18 (YA)	cTBS for 40 s (600 pulses) at 80% RMT	Offline with 3 sessions separated by at least 1 day prior to encoding Within subjects	Encoding of fractal objects and memory awareness judgments	sMRI and 10–20 system	Frontopolar cortex (i.e., MNI: ± 29, 66, 10) and DLPFC (i.e., MNI: ± 52, 15, 29)	Active vertex (Paracentral lobule; MNI: ± 4,−42, ± 73; Cz)	Fractal-object associative-recognition and memory awareness judgements	↑ in memory judgements and confidence ratings for frontopolar cortex compared to DLPFC and control. No differences in accuracy following either stimulation.
^*^Yazar et al., 2014	69 (YA)	cTBS for 40s (600 pulses) at 70% RMT	Offline immediately after encoding and prior to retrieval Between subjects	Retrieval of auditory words (male vs. female voice)	sMRI	Left Angular Gyrus (-i.e., MNI: 43, −66,38), left intraparietal sulcus (i.e., MNI: −38, −62,46).	Active vertex (i.e., MNI: 0, −15, 74)	Recognition, confidence ratings, and source recollection of words. Free and cued recall and confidence ratings for words	↓ in source recollection confidence following left angular gyrus stimulation compared to vertex. No differences in memory performance.
^*^Yazar et al., 2017	23 (YA)	cTBS for 40 s at 70% RMT	Offline immediately after encoding prior to retrieval with 2 sessions separated by 3 days Within Subjects	Retrieval of audiovisual information	Estimated MRI	Left angular gyrus (i.e., MNI: −43, −66, 38)	Active vertex (i.e., MNI: 0, 15, 74)	Recognition and reaction time. Single, multi-modal, and cross-modal source recollection	↓ in cross-modal source recollection following angular gyrus stimulation compared to control. No differences for recognition or source accuracy or reaction times.
^*^Bonnici et al., 2018	22 (YA)	cTBS for 40 s at 70% RMT	Offline prior to retrieval for two sessions separated by 1 week Within subjects	Retrieval of autobiographical and word pair associations	sMRI	Left angular gyrus (MNI: −43, −66, 38)	Active vertex (0, −15, 74)	Free and cued recall of autobiographical memories and free and cued recall of word pairs	↓ in free recall of autobiographical memories after left angular gyrus stimulation compared to control. ↓ in first-person perspective memory. following left angular gyrus cTBS compared to control. No differences in cued recall of autobiographical memories. No differences in free or cued recall of word pairs.
^*^Bonnì et al., 2015	30 (YA)	cTBS for 40 s (600 pulses) at 100% MT	Offline 15 min after encoding and prior to retrieval Between subjects	Retrieval of words and color context	sMRI	Precuneus (i.e., MNI: 0, −67± 3, 38 ± 10) or Posterior parietal cortex (i.e., MNI: −46 ± 7, −66 ± 5, 47 ± 3)	Active vertex (i.e., Cz)	Associative recognition	↑ in context retrieval following precuneus stimulation (i.e., ↓ in source memory errors).
^*^Demeter et al., 2016 Exp 1	16 (YA)	Short iTBS for 2 s trains at 80% AMT	Online 700, 2,900, and 5,100 ms Within subjects	Deep encoding of word pairs	10–20 system	Left DLPFC (i.e., F3)	Active vertex (i.e., Cz)	Recognition of words and confidence rating	↑ in word recognition and confidence following left DLPFC stimulation compared to control and persisted for items encoded up to 5 s following stimulation. No differences in RT.
^*^Demeter et al., 2016 Exp 2	17 (YA)	Short iTBS for 2 s trains at 80% AMT	700 ms, 5 s, 7 s, 11 s, 15 s. prior to stimulus onset Within subjects	Deep encoding of word pairs	10–20 system	Left DLPFC (i.e., F3)	Active vertex (i.e., Cz)	Recognition of words and confidence rating	↑ in word recognition and confidence following left DLPFC stimulation compared to control and persisted for items encoded up to 15 s following stimulation. No differences in RT.
Škrdlantová et al., 2005	10 (YA)	0.9 Hz rTMS at 110% MT	Online rTMS application began 1 min before test and continued through the acquisition phase Between subjects	Encoding of words and faces	Distance measurements	Left DLPFC	Sham to left DLPFC with coil angled away	Recognition of words and faces	↓ free recall of words. No difference in face recognition.
Innocenti et al., 2013	13 (YA)	10 Hz rTMS for 500 ms at 90% MT	Online Stimulation 100 ms before offset of word presentation Between subjects	Encoding of words	sMRI	Left DLPFC and left IPL	Active vertex and no TMS	Free recall of words	Left DLPFC: ↓ in primacy effect, retained recency effect. Left IPL: ↓ in recency effect, retained primacy effect. Both control conditions showed retained primacy and recency effects.
^*^Wang et al., 2014	16 (YA)	20 Hz rTMS for 20 min (1,600 pulses) at 100% MT (measured via EMG)	Offline for 5 days/stimulation site Within subjects	Cannot be distinguished	sMRI and fMRI	Cortical-hippocampal brain network via the left parietal cortex (inferior parietal lobule; nearest to MNI: −47, −68, 36)	Sham to left parietal cortex with a spacer	Cued-recall (face-name pairs)	↑ in associative memory performance following multi-session stimulation.
^*^Hawco et al., 2017	17 (YA)	10 Hz rTMS at 100% RMT	Online onset (i.e., 200, 600, 1,000 ms) Within subjects	Associative encoding of object pairs (related vs. unrelated)	10–20 system	DLPFC (i.e., F3)	No TMS	Cued recall	↑ in memory for 600 ms object stimulation offset compared to no rTMS for related pairs. ↓ in memory for 1,000 ms object stimulation offset compared to no rTMS for related pairs.
^*^Hanslmayr et al., 2014	19 (YA)	18.7, 10.7, or 6.8 Hz rTMS at 90% RMT	Online 0.5 s after item onset Within subjects	Encoding of words	sMRI	Left inferior frontal gyrus (MNI: −48, 9, 30)	Active at and 10.7, 6.8 Hz. Sham with coil angled away at 18.7 Hz	Free recall of words	↓ in word encoding following beta (18.7 Hz) entrainment to the left inferior frontal gyrus.
Balconi and Ferrari, 2013	28 (YA)	5 Hz rTMS at 100% MT	Online for 1 s during retrieval phase Within subjects for TMS condition (F3, Cz, or sham) Between subjects for high vs. low anxiety subjects	Retrieval of emotional words	Estimated MRI	Left DLPFC (i.e., F3)	Active vertex and sham with coil angled away	Recognition	High anxiety: ↑ recall performance following DLPFC stimulation compared to active and sham controls. Low anxiety: ↓ recall performance following DLPFC stimulation compared to active and sham controls.
^*^Tambini et al., 2018	22 (YA)	cTBS at 80% AMT (600 pulses/session)	Offline prior to encoding and for 3 sessions over 3 weeks Within subjects	Encoding of objects and spatial locations	sMRI and fMRI	Hippocampus via functionally connectivity with right posterior inferior parietal cortex (pFPC; MNI: 43, −67, 28)	Active control (i.e., primary somatosensory cortex) and no TMS	Recognition, spatial recall, and confidence ratings	↑ in associative memory and confidence ratings following cTBS to right pIPC compared to control and no TMS. Hippocampal—pIPC functional connectivity predicted memory benefits. No differences in item memory.
^*^Nilakantan et al., 2017	12 (YA)	20 Hz for 20 min (1,600 pulses a session) rTMS at 100% MT	Offline across 5 days/week for 2 weeks separated by 4 weeks Within subjects	Cannot be distinguished	sMRI and fMRI	Posterior cortical-hippocampal network via functional connectivity of the left parietal cortex (i.e., MNI: −47, −68, 36)	Sham, vertex (i.e., MNI: 0, −42, 73) and no TMS baseline	General and precision spatial recollection task	↑ in precision recognition following 5 days of left parietal cortex stimulation compared to control (sham and no TMS collapsed). No differences in general recognition.
^*^Waldhauser et al., 2016 Exp 2.	23 (YA)	17.5 Hz rTMS at 90% PT	Online during retrieval from 33.5 to 204.5 ms after cue presentation Within subjects	Retrieval of pictures	sMRI	Left and right lateral occipital cortex (i.e., MNI: ± 40,−78, 0)	Sham to lateral occipital cortex with coil angled away	Item recognition of pictures and source memory spatial locations	↓ in source memory compared to control. No differences in item recognition memory compared to control.
Julian et al., 2016	12 (YA)	cTBS at 3 pulse bursts of 50 Hz repeated every 200 ms for 40 s 80% phosphene threshold	Online Two sessions separated by 1 week, with stimulation order counterbalanced across subjects Within subjects	Encoding of spatial associations	sMRI and fMRI	Right occipital place area	Active vertex	Spatial navigation with reference to boundaries	↓ in accurate navigation to boundary-tethered objects, but not landmark-tethered objects.
**OLDER ADULTS**									
Rektorova et al., 2005	7 (OA)	10 Hz rTMS at 100 % RMT	Offline, neuropsychological testing on day 0, 1, and 4 pre- and post- TMS session Within subjects	Cannot be distinguished	Not stated	Left DLPFC	Active control (i.e., motor cortex)	Rey-Osterrieth Complex Figure Test (RCFT), story subtest of the Wechsler Memory Scale (WMS)	No significant difference between stimulation and baseline for memory psychological tests following stimulation to the left DLPFC or left motor cortex.
Manenti et al., 2011	38 (OA with cerebrovascular disease)	20 Hz (640 total pulses) rTMS at 90% RMT	Online at word presentation for 450 ms Within subjects	Encoding or retrieval of word pairs	Estimated Talairach	Right DLPFC (Talairach: 36, 37, 39) & left DLPFC (i.e., Talairach: −36, 37, 39)	Sham coil to left DLPFC during encoding and right DLPFC during retrieval	Word-pair retrieva	Low-performing: Encoding: ↓ word-pair retrieval, left more than right DLPFC. Retrieval: ↓ word-pair retrieval right same as left DLPFC. High-performing: Encoding: ↓ word-pair retrieval, right same as left DLPFC. Retrieval: ↓ word-pair retrieval right same as left DLPFC.
Turriziani et al., 2012 Exp 4	8 (OA with MCI)	1 Hz rTMS at 90% MT	Offline with 2 sessions separated by 6 h during 10 min delay between encoding and recognition Within subjects (real vs. sham TMS) and Between subjects (left vs. right DLFPC)	Nonverbal retrieval of faces, buildings, or word.	10–20 system	Right DLPFC (i.e., F4) or left DLPFC (i.e., F3)	Sham with coil angled away	Recognition of faces, buildings, or words	↑ in memory performance for older adults with MCI with 1 Hz stimulation to right DLPFC compared to sham.
Peña-Gomez et al., 2012	20 (OA; 9 APOE carriers, 11 non- carriers)	5 Hz rTMS for 5 min (5,000 total pulses) at 80% MT	Offline prior to encoding Between subjects	Encoding of face-name associations	Distance measurements	Prefrontal cortex (interhemispheric fissure)	Non-APOE carriers	Recognition of face-name associations	↑ in memory performance for both carriers and non-carriers.
Solé-Padullés et al., 2006	39 (OA with memory dysfunction)	5 Hz rTMS at 80% MT	Offline prior to encoding Between subjects	Encoding of face-name associations	Distance measurements	Prefrontal cortex (5 cm anterior of interhemispheric fissure).	Sham with coil angled away	Recognition of face-name associations	↑ memory performance following PFC 5 Hz stimulation.
Drumond Marra et al., 2015	34 (OA with MCI)	10 Hz rTMS (2,000 pulses/session) at 110% MT	Offline for 10 consecutive weekdays Between subjects	Encoding and retrieval of everyday memory tasks	Distance measurements	Left DLPFC	Sham coil	The Rivermead behavioral memory test	↑ in episodic memory following 10 session of left DLPFC stimulation compared to sham.
Rossi et al., 2004	29 (OA)	20 Hz rTMS at 90% MT	Online during picture presentation for 500 ms Within subjects	Encoding or retrieval of pictures	10–20 system	Right DLPFC (i.e., F4) or left DLPFC (i.e., F3)	Sham with coil angled away and no TMS	Recognition of pictures	Encoding: ↓ picture retrieval, left more than right DLPFC. Retrieval: ↓ picture retrieval, right more than left DLPFC.
Cotelli et al., 2012	1 (OA with aMCI) 22 (healthy controls)	20 Hz rTMS at 100% MT.	Offline 50 trains, 2,000 pulses/session, five sessions/week for 2 weeks Between subjects	Cannot be distinguished	sMRI	Left parietal cortex (IPL; Talairch: −44, −51, 43)	No TMS healthy control group	Recognition of face-name pair associations	↑ associative memory for aMCI patient at 2 weeks and 24 weeks compared to baseline.
Vidal-Piñeiro et al., 2014	24 (OA)	iTBS (600 total pulses) at 80% AMT	Offline prior to encoding Between subjects (TMS vs. sham) and within (TMS and no TMS)	Deep and shallowing encoding of verbal words	sMRI	Left inferior frontal gyrus	Sham coil and no TMS	Recognition of verbal words	No differences in memory accuracy. ↑ in activation during semantic processing in posterior occipital and cerebellar areas for deep encoding following stimulation to left inferior frontal gyrus.
Koch et al., 2018	14 (OA with prodromal Alzheimer disease)	20 Hz rTMS for 1,600 pulses/session at 100% RMT/AdjMT	Offline with 10 sessions (5 session/week) with 2 week separation between cross over stimulation. Within subjects	Encoding of auditory verbal information	sMRI	Precuneus	Sham coil and active control (left posterior parietal cortex)	Delayed and immediate recall of auditory verbal information	↑ in delayed recall following precuneus stimulation. No differences in immediate recall.
Davis et al., 2017	14 (OA)	1 or 5 Hz rTMS for 10 min (600 total pulses) at 120% MT (measured via EMG)	Offline prior to encoding task. Within subjects	Encoding of concrete words and objects from sentences	sMRI	Left DLPFC (i.e., left middle frontal gyrus point of greatest activation for successful encoding)	N/A	Recognition of word pairs	No differences in memory performance between 1 and 5 Hz rTMS. ↑ in SME and local increases in PFC connectivity following 5 Hz stimulation. ↑ left and right PFC connectivity and to a lesser degree increases in bilateral parietal areas. connectivity following 1 Hz stimulation.
**MIXED AGE CLINICAL POPULATIONS**									
Kavanaugh et al., 2018	84 (18–70, M = 48 and 46)	10 Hz rTMS at 120% RMT	Offline Between subjects	None; treatment for depression	Anatomical landmarking	Left DLPFC and bilateral dorsomedial prefrontal cortex	Sham coil	Immediate and delayed word recall. Recognition of words and pictures	↑ in episodic memory following left DLPFC stimulation compared to sham.
Baudic et al., 2013	38 (18+, M = 52 and 50)	10 Hz rTMS at 80% RMT	Offline Neuropsychological testing after 7 rTMS sessions and 11 sessions (week 3 and week 11) Between subjects	Cannot be distinguished; treatment for pain associated with fibromyalgia	Not stated	Left primary motor cortex	Sham coil	Rey Auditory Verbal Learning Test	No differences in memory performance following left primary motor cortex stimulation compared to sham.
Qiao et al., 2016	38 (18+)	10 Hz rTMS at 80% RMT	Offline Between subjects	None; treatment for alcohol-dependent patients	Not stated	Right DLPFC	Sham coil placed perpendicular to scalp	Hopkins Verbal Learning Test-Revised (HVLT-R), Brief Visuospatial Memory Test-Revised (BVMT-R)	↑ memory performance following rTMS stimulation of the right DLPFC in patients recovering from alcohol dependency.

* *Denotes experiments that contributed effect sizes in the meta-analysis*.

### Quality Assessment

**Figure 2:**
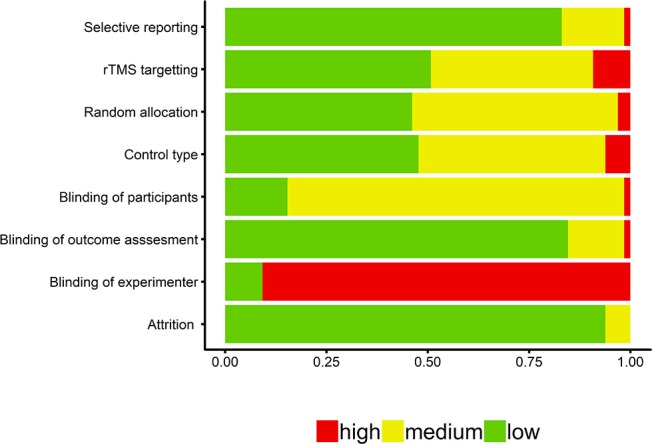
Quality assessment findings for the 59 articles included in the systematic review. Blinding of outcome assessment reflects blinding of the experimenter in coding memory outcome measures (e.g., recall tasks).

The proportion of studies classified as low, medium, and high risk for the different categories of the quality assessment analysis are presented in Figure 2. Overall, many of the studies implemented appropriate methodological protocols for random allocation of participants to conditions, blinding of the experimenters assessing effects on the outcome measure(s), and non-selective reporting of outcome measures. However, several studies either did not implement proper blinding of participants and/or experimenters, or did not provide enough information to confirm proper blinding procedures. There was low concern for the degree of participant attrition that was reported. For our additional quality assessment measure of control type, we found that the majority of studies implemented either active or sham controls. With regards to the rTMS targeting procedure, we found that the majority of studies implemented either low risk neuronavigational procedures or used the 10–20 system for targeting cortical regions of interest.

### Evaluation of Publication Bias

**Figure 3:**
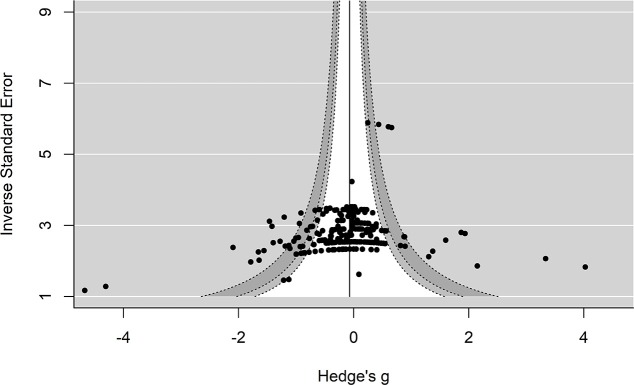
Contour-Enhanced funnel plot. Reference line is set to *g* = −0.06. Contour lines represent *p*-value significance (i.e., <0.05, < 0.01) with the white area representing *p*-values > 0.05.

Several approaches were used to examine possible publication bias. First, we examined the enhanced-contour plot (i.e., effect size vs. 1/standard error). The reference line is set to *g* = −0.06 with the contour shading representing *p*-value significance. The enhanced-contour plot revealed some asymmetry, but no systematic patterns of missing non-significant effect sizes, see Figure 3. Given the numerous effect sizes collected, the larger effect sizes observed in the funnel plots are expected. Importantly, they appear to be symmetrically distributed. An examination of influential points revealed no Cook's d values above 0.5.

**Figure 4:**
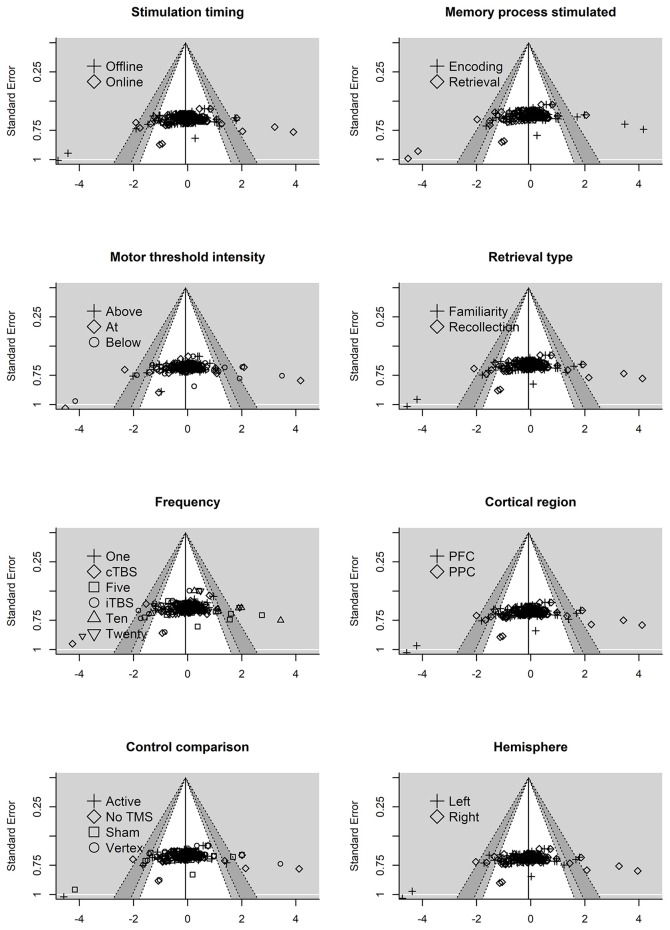
Residual funnel plots for moderator variables. The residuals are on the x-axis with the standard error on the y-axis. Contour lines represent *p*-value significant (i.e., <0.05, < 0.01) with the white area representing *p*-values > 0.05. Reference line set to *g* = −0.06.

Next, we examined the residual plots (residuals vs. standard error) for each moderator variable, see Figure 4. The reference line is set to average effect size with contour shading once again representing *p*-value significance. The residual plots revealed some asymmetry across the moderator variables.

Interpreting funnel plots is inherently difficult (Terrin et al., 2005) and may be further complicated in three-level models (e.g., observed asymmetry may not be problematic). For example, clustered data points due to the three-level structure could potentially be misinterpreted as bias. Therefore, in a more formal test of asymmetry we entered standard error into the model. This revealed no significant moderating effect of standard error on effect size, *F*_(1, 244)_ = 1.76, *p* = 0.18, indicating that the funnel plot was not significantly asymmetrical. Collectively, this suggests that publication bias is unlikely to be problematic in this analysis. However, future research is needed to properly address how to handle detecting and correcting for missing data (e.g., publication bias) in three-level models.

### Overall Relationship Between rTMS and Episodic Memory

**Figure 5:**
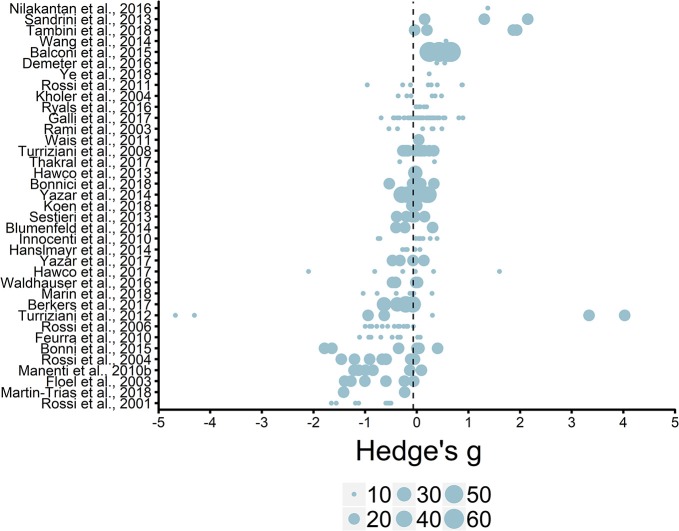
Overview of effect sizes included in meta-analysis. Reference line set to the average effect size (*g* = −0.06). Legend represents study sample size ranges: 10–19, 20–29, 30–39, 40–49, 50–59, 60–69.

We initially entered individual effect sizes (i.e., Effect Size ID) and studies (i.e., Study ID) as random effects. This model revealed the average effect size (g = −0.06, *SE* = 0.08) was not significantly different from zero, *t*_(244)_ = −0.75, *p* = 0.45, see Figure 5. Thus, we do not see an overall effect of rTMS on episodic memory performance, which is unsurprising given that most studies selected and implemented protocol parameters to either enhance or impair episodic memory functioning.

### Heterogeneity of Effect Sizes

We tested the heterogeneity of effect sizes to examine if differences between effect sizes were systematic and not due to random sampling variance. If a substantial amount of between- or within-study effect size variance is found, potential moderators can be added into the model to explain this variability. There was significant within-study variance (level 2), estimate = 0.19, *p* < 0.01 and between-study variance (level 3), estimate = 0.12, *p* < 0.01, demonstrating that effect sizes varied both within and between studies. Consequently, 44.58% of the variance is accounted for by differences in effect sizes within studies, 27.25% by variance between studies, and 28.07% by random sampling variance. The significant variability across both levels highlights the substantial differences in effect sizes between studies and within the same study. Therefore, we next conducted a moderation analysis in order to assess whether the observed differences in effect sizes were moderated by other factors.

### rTMS Parameter Moderator Analysis

A moderation test revealed a significant moderating effect of rTMS frequency on episodic memory effects, *F*_(6, 224)_ = 2.40, *p* = 0.03, see Table 2. Specifically, 1 Hz (β = 0.59, *SE* = 0.20) rTMS protocols led to significantly larger enhancing effects compared to 10 Hz, (β = −0.28, *SE* = 0.20), 20 Hz (β = −0.22, *SE* = 0.14), iTBS (β = −0.42, *SE* = 0.36), and cTBS (β = −0.05, *SE* = 0.15). The comparisons between other frequencies were not significant. The aggregate effects of stimulation intensity or timing, or the targeted hemisphere, cortical region, memory process, or retrieval type, or the type of control condition were not significant moderators of rTMS effect sizes, see Table 2.

**Table 2:** Main effects of moderator variables (F-values) and beta and t values of each level in the meta-analysis.

**Moderator variables and levels**	**^**#**^ of studies**	**^**#**^ of effect sizes**	**β_**0**_ (*SE*)**	***t_**0**_***	**β_**1**_ (*SE*)**	***t_**1**_***	***F***	**Within study variance (level 2)**	**Between study variance (level 3)**
**Standard error**		245	−0.28 (0.21)	−1.33			*F*_(1, 244)_ = 1.76		
**Stimulation timing**		245					*F*_(2, 243)_ = 2.15	0.18 (42.72%)	0.12 (28.21%)
Online	23	176	−0.19 (0.10)	**–**1.84^*^	−30 (0.16)	−1.91^*^			
Offline (RC)	19	69	0.12 (0.12)	0.96					
**Frequency**		230					*F*_(6, 224)_ = 2.40^*^	0.16 (37.67%)	0.14 (33.59%)
1 Hz (RC)	7	13	0.59 (0.20)	2.88^**^					
5 Hz	2	8	0.18 (0.30)	0.62	−41 (0.32)	−1.27			
10 Hz	6	35	−0.28 (0.20)	−1.42	−87 (0.28)	−3.06^**^			
20 Hz	12	116	−0.22 (0.14)	−1.63	−81 (0.25)	−3.30^***^			
iTBS	3	5	−0.42 (0.36)	−1.55	−1.00 (0.42)	−2.42^*^			
cTBS	9	53	−0.05 (0.15)	−0.36	−61 (0.25)	−2.38^*^			
**Motor threshold intensity (MTI)**		227					*F*_(3, 224)_ = 1.76	0.20 (43.41%)	0.14 (30.18%)
Above 100% MTI (RC)	4	21	−0.33 (0.22)	−1.51					
At 100% MTI	10	35	0.23 (0.17)	1.36	0.56 (0.28)	2.03^*^			
Below 100% MTI	26	171	−0.15 (0.10)	−1.48	0.18 (0.21)	0.86			
**Hemisphere**		227					*F*_(2, 225)_ = 0.96	0.22 (47.44%)	0.13 (26.57%)
Left (RC)	34	169	−0.07 (0.09)	−0.75					
Right	16	58	0.07 (0.12)	0.61	0.14 (0.11)	1.34			
**Cortical region**		237					*F*_(2, 235)_ = 0.45	0.21 (45.73%)	0.13 (27.46%)
Frontal cortex (RC)	29	175	−0.09 (0.10)	−92					
Parietal cortex	13	62	0.03 (0.15)	0.19	0.12 (0.18)	0.67			
**Memory process stimulated**		240					*F*_(2, 238)_ = 1.51	0.14 (37.74%)	0.12 (30.21%)
Encoding (RC)	24	159	−0.12 (0.09)	−1.35					
Retrieval	18	81	−0.14 (0.10)	−1.44	−0.02 (0.11)	−0.22			
**Retrieval type**		245					*F*_(2, 243)_ = 0.78	0.19 (44.03%)	0.12 (27.69%)
Recollection	22	79	−0.01 (0.10)	−0.01	0.10 (0.11)	0.99			
Familiarity (RC)	30	166	−0.10 (0.09)	−1.15					
**Control comparison**		245					*F*_(4, 241)_ = 0.51	0.20 (44.74%)	0.12 (27.62%)
Active (RC)	18	37	−0.14 (0.12)	−1.16					
Active vertex	22	79	−0.01 (0.10)	−0.04	0.14 (0.12)	1.17			
Sham	16	65	−0.09 (0.11)	−0.83	0.04 (0.12)	0.34			
No TMS	11	64	−0.07 (0.11)	−0.67	0.06 (0.12)	0.50			

# *of studies represents the number of studies that contributed an effect size. # of effect sizes represents the number of effect sizers. Reference category for moderator comparisons are denoted by RC. β_0_ values represent estimated mean effect size individual moderator levels. t_0_ t values for individual moderator variable effect size means being significantly different from zero. β_1_ represents the estimated regression coefficient. t_1_ represents t values for individual moderator variables comparison to reference category. F-values represent the omnibus test of all coefficients in the model for each moderator. Level 2 is the variance attributed to all the moderators that varies within studies. Level 3 is the variance attributed to between study differences*.

* p < 0.05,

** p < 0.01,

*** *p < 0.001*.

### Interactions

#### Effect of Frequency and Motor Threshold Intensity

Although we did not find support for MTI moderating episodic memory performance overall, we anticipated that MTI may interact with frequency. Recent findings have shown that varying MTI's during iTBS follow an inverted U-shaped pattern in modulating working memory performance (Chung et al., 2018). An investigation of MTI (above, at, or below) and frequency (1, 5, 10, 20, cTBS, iTBS) revealed an interaction, *F*_(14, 202)_ = 1.78, *p* = 0.05, see Tables 3, 4. In order to examine what was driving this interaction we considered how differences between MTI (above vs. at vs. below) at each frequency affected episodic memory. However, the number of comparisons were restricted due to the limited number of studies that included frequencies at different MTIs. Follow-up tests revealed that the advantage of 1 Hz rTMS compared to 10, 20, iTBS, and cTBS protocols in producing significantly stronger enhancing effects of experimental/treatment rTMS vs. control/sham rTMS on memory performance was specific to intensities below MTI. There was also a significant effect of 20 Hz rTMS “below” MTI (β = −0.38, *SE* = 0.17) in that it produced a larger negative effect of experimental/treatment rTMS vs. control/sham rTMS compared to 20 Hz “at” MTI (β = 0.40, *SE* = 0.35), *F*_(1, 202)_ = 3.98, *p* = 0.04. There were no other differences between levels of MTI for any other frequency, see Tables 3–5,^1^.

**Table 3:** Descriptive statistics for motor threshold intensity by frequency interaction.

	**Frequency**						
**Motor threshold intensity**		**1**	**5**	**10**	**20**	**iTBS**	**cTBS**
**ABOVE**							
	β (*SE*)	0.40 (0.57)		−0.50 (0.47)	−0.77 (0.32)		
	n	3		10	8		
	k	2		1	1		
	CI	[−73, 1.53]		[−1.42, 0.13]	[−1.39, −0.14]		
**AT**							
	β (*SE*)	0.58 (0.34)	0.48 (0.48)	−0.13 (0.39)	0.40 (0.35)		−0.49 (0.50)
	n	4	4	7	8		6
	k	3	1	2	3		1
	CI	[−0.08, 1.24]	[−0.47, 1.44]	[−0.89, 0.63]	[−0.29, 1.09]		[−1.47, 0.49]
**BELOW**							
	β (*SE*)	0.92 (0.41)	−0.12 (0.51)	−0.28 (0.29)	−0.38 (0.17)	−0.44 (0.38)	0.01 (0.17)
	n	4	4	18	88	5	47
	k	2	1	3	8	3	8
	CI	[0.10, 1.75]	[−1.12, 0.88]	[−0.86, 0.29]	[−0.71, −0.04]	[−1.19, 0.31]	[−0.34, 0.34]

*Means, standard errors, and confidence intervals. The number of studies and effect sizes are denoted by k and n, respectively. Given the available studies not all possible combinations were represented*.

**Table 4:** Comparisons for frequency by motor threshold intensity interaction.

	**Motor threshold intensity**		
**Comparisons**	**Below**	**At**	**Above**
1 vs. 5	*F*_(1, 202)_ = 2.54, *p* = 0.11	*F*_(1, 202)_ = 0.06, *p* = 0.80	
1 vs. 10	*F*_(1, 202)_ = 5.57, *p* = 0.02^*^	*F*_(1, 202)_ = 1.93, *p* = 0.16	*F*_(1, 202)_ = 1.47, *p* = 0.23
1 vs. 20	*F*_(1, 202)_ = 8.25, *p* < 0.01^**^		*F*_(1, 202)_ = 3.18, *p* = 0.08
1 vs. cTBS	*F*_(1, 202)_ = 4.17, *p* = 0.04^*^		
1 vs. iTBS	*F*_(1, 202)_ = 5.80, *p* = 0.02^*^		
5 vs. 10	*F*_(1, 202)_ = 0.05, *p* = 0.81	*F*_(1, 202)_ = 1.09, *p* = 0.30	
5 vs. 20	*F*_(1, 202)_ = 0.22, *p* = 0.64		
5 vs. cTBS	*F*_(1, 202)_ = 0.05, *p* = 0.82		
5 vs. iTBS	*F*_(1, 202)_ = 0.24, *p* = 0.62		
10 vs. 20	*F*_(1, 202)_ = 0.08, *p* = 0.78	*F*_(1, 202)_ = 1.06, *p* = 0.30	*F*_(1, 202)_ = 0.23, *p* = 0.63
10 vs. cTBS	*F*_(1, 202)_ = 0.68, *p* = 0.41		
10 vs. iTBS	*F*_(1, 202)_ = 0.10, *p* = 0.75		
20 vs. cTBS	*F*_(1, 202)_ = 2.36, *p* = 0.13		
20 vs. iTBS	*F*_(1, 202)_ = 0.02, *p* = 0.89		
iTBS vs. cTBS	*F*_(1, 202)_ = 1.09, *p* = 0.30		

Only available frequency comparisons are listed that contained observable data for at least one of the motor threshold levels. Empty cells represent no observed effect sizes for comparison

* p < 0.05,

** *p < 0.01*.

**Table 5:** Additional comparisons for frequency by motor threshold intensity interaction.

	**Frequency**					
**Comparison**	**1**	**5**	**10**	**20**	**iTBS**	**cTBS**
Above vs. At	*F*_(1, 202)_ = 0.01, *p* = 0.99		*F*_(1, 202)_ = 0.37, *p* = 0.54	*F*_(1, 202)_ = 6.12, *p* = 0.01^*^		
Above vs. Below	*F*_(1, 202)_ = 0.54, *p* = 0.46		F_(1, 202)_ = 0.15, *p* = 0.69	*F*_(1, 202)_ = 6.12, *p* = 0.01^*^		
At vs. Below	*F*_(1, 202)_ = 0.92, *p* = 0.34	*F*_(1, 202)_ = 0.75, *p* = 0.38	*F*_(1, 202)_ = 0.10, *p* = 0.75	*F*_(1, 202)_ = 3.98, *p* = 0.04^*^		*F*_(1, 202)_ = 0.86, *p* = 0.35

Empty cells represent no observed effect sizes for comparison

* *p < 0.05*.

#### Effect of Frequency and Stimulation Timing

There was a significant interaction of stimulation timing (online vs. offline) and frequency (1, 5, 10, 20, iTBS, cTBS) on episodic memory effects, *F*_(9, 221)_ = 4.16, *p* < 0.01. We first examined differences between frequencies within online and offline stimulation protocols. Subsequently, we looked at differential outcomes between online vs. offline protocols for specific frequencies. However, the number of comparisons we could make was limited due to the small number of studies that included online or offline protocols at each frequency.

Follow-up tests revealed that during online stimulation protocols, 1 Hz rTMS (β = 0.60, *SE* = 0.45) compared to 20 Hz rTMS (β = −0.32, *SE* = 0.12) led to larger enhancing effects of experimental/treatment rTMS vs. control/sham rTMS on memory performance, see Tables 6, 7. No other interactions with online stimulation were significant.

**Table 6:** Descriptive statistics for stimulation timing by frequency interaction.

	**Frequency**						
**Stimulation timing**		**1**	**5**	**10**	**20**	**iTBS**	**cTBS**
**ONLINE**							
	β (*SE*)	0.60 (0.45)	0.20 (0.28)	−0.28 (0.17)	−0.32 (0.12)	0.46 (0.43)	
	n	2	8	35	114	2	
	k	1	2	6	10	2	
	CI	[−0.29, 1.49]	[−0.35, 0.75]	[−0.61, 0.05]	[−0.56, −0.08]	[−0.38, 1.31]	
**OFFLINE**							
	β (*SE*)	0.58 (0.21)			0.94 (0.45)	−1.84 (0.53)	−0.06 (0.13)
	n	11			2	3	53
	k	7			2	1	9
	CI	[0.18, 0.99]			[0.05, 1.82]	[−2.89, −0.79]	[−0.31, 0.20]

*Means, standard errors, and confidence intervals. The number of studies and effect sizes are denoted by k and n, respectively. Given the available studies not all possible combinations were represented*.

**Table 7:** Stimulation timing moderator comparisons.

	**Stimulation timing**	
**Comparisons**	**Online**	**Offline**
1 vs. 5	*F*_(1, 221)_ = 0.86, *p* = 0.35	
1 vs. 10	*F*_(1, 221)_ = 3.39, *p* = 0.07	
1 vs. 20	*F*_(1, 221)_ = 3.93, *p* = 0.04^*^	*F*_(1, 221)_ = 0.51, *p* = 0.48
1 vs. iTBS	*F*_(1, 221)_ = 0.05, *p* = 0.82	*F*_(1, 221)_ = 17.98, *p* < 0.01^***^
1 vs. cTBS		*F*_(1, 221)_ = 6.85, *p* < 0.01^**^
5 vs. 10	*F*_(1, 221)_ = 2.20, *p* = 0.14	
5 vs. 20	*F*_(1, 221)_ = 2.95, *p* = 0.08	
5 vs. iTBS	*F*_(1, 221)_ = 0.26, *p* = 0.61	
10 vs. 20	*F*_(1, 221)_ = 0.04, *p* = 0.85	
10 vs. iTBS	*F*_(1, 221)_ = 2.63, *p* = 0.11	
20 vs. iTBS	*F*_(1, 221)_ = 3.13, *p* = 0.08	*F*_(1, 221)_ = 15.87, *p* < 0.01^***^
20 vs. cTBS		*F*_(1, 221)_ = 4.50, *p* = 0.03^*^
iTBS vs. cTBS		*F*_(1, 221)_ = 10.58, *p* < 0.01^**^

*Only available frequency comparisons are listed that contained observable data for either online or offline protocols. Empty cells represent no observed effect sizes for comparison*.

* p < 0.05,

** p < 0.01,

*** *p < 0.001*.

Following offline stimulation protocols, 1 Hz rTMS (β = 0.58, *SE* = 0.21) led to significantly larger enhancing effects compared to both iTBS (β = −1.84, *SE* = 0.53) and cTBS (β = −0.06, *SE* = 0.13). In addition, offline 20 Hz rTMS (β = 0.94, *SE* = 0.45) led to significantly larger enhancing effects compared to both iTBS and cTBS. Interestingly, offline iTBS led to significantly larger negative effects compared to cTBS. No other comparisons were significant, although a full decomposition of the interaction was not possible because the number of studies with offline stimulation for each type of frequency were limited, see Table 7.

Additional follow-up tests revealed that stimulation timing significantly influenced the directionality of the effects of 20 Hz rTMS. Specifically, online 20 Hz rTMS led to larger negative effects, while offline 20 Hz rTMS led to larger enhancing effects of experimental/treatment rTMS vs. control/sham rTMS on memory performance, *F*_(1, 221)_ = 7.33, *p* < 0.01. There were no other significant differences.

#### Memory Processes and Hemispheric Stimulation

Findings of individual studies have reported that there may be different effects of rTMS when stimulating the left or right hemisphere during either encoding or retrieval. To assess potential aggregate effects across studies, we examined the interaction between stimulated hemisphere (left vs. right hemisphere) and memory process stimulated (encoding vs. retrieval processes). However, there was no significant interaction between the effect sizes of stimulating different memory processes and hemisphere, *F*_(4, 218)_ = 1.03, *p* = 0.39.

#### Retrieval Type and Cortical Regions

Although it is plausible that the effects of stimulating either frontal or parietal cortex may differ for familiarity- vs. recollection-based retrieval processes, we found no significant interaction between effect sizes of the stimulated cortical region (FC vs. PC) and retrieval type (familiarity vs. recollection), *F*_(4, 233)_ = 0.45, *p* = 0.77, although, again, inferences are currently limited by the relatively small numbers of studies that could be included in such a moderation-based meta- analysis.

## Discussion

A systematic review of the literature spanning the past 30 years yielded 59 articles that focused on how TMS can modulate episodic memory in younger and older adults. To better understand the circumstances of when rTMS will enhance or impair memory, we conducted a three-level random effects meta-analysis of 245 effect sizes aggregated from 37 articles focused on young adults. Because the effects of some studies used parameters designed to impair memory to probe the necessity of a stimulated brain region in a particular process relevant for episodic memory, whereas others implemented designs to facilitate episodic memory, the mean effect size, collapsing across all parameters is relatively uninformative.

### Enhancing Effects of 1 Hz rTMS

Moderation analyses revealed that stimulation frequency modulated the effect of rTMS on episodic memory in younger adults, with larger facilitatory effects of 1 Hz (*g* = 0.59) rTMS compared to 10 (*g* = −0.28), 20 (*g* = −0.22), iTBS (*g* = −0.42), and cTBS (*g* = −0.05) protocols. The facilitatory effect of 1 Hz rTMS may appear counter-intuitive because low frequency (e.g., <1 Hz) rTMS is typically thought to lead to inhibitory effects on neural processing of targeted regions, whereas, many high frequency protocols are generally thought to lead to enhancing cortical excitability (Walsh and Cowey, 2000; Rossi and Rossini, 2004). Relatedly, the numerically larger negative effects of iTBS compared to cTBS paradigms is also surprising given that cTBS is typically assumed to have long-term-depression-like inhibitory effects (LTD) that can suppress cortical excitability in targeted regions whereas iTBS is assumed to lead to long-term potentiation-like effects (LTP) that can enhance cortical excitability (Huang et al., 2005). However, these findings stem from physiological studies that modulated cortical activity in the motor cortex (Jelić et al., 2015), while the studies in the present meta-analysis generally targeted prefrontal and parietal regions. One possible explanation is that the effects of TMS may differ according to the structural and functional architecture of the targeted region (e.g., motor cortex vs. prefrontal/parietal cortex). However, strong inferences based on these findings are currently limited due to the numerous differences between studies in the specific rTMS parameters and experimental designs that were used, which may interact to affect episodic memory.

### Interactions Between rTMS Frequency and Stimulation Intensity

Interaction analyses revealed facilitatory effects of 1 Hz rTMS for both online and offline stimulation. However, the effects of 1 Hz rTMS was driven by stimulating below MTI. While the underlying mechanisms of 1 Hz rTMS on episodic memory are poorly understood, one possible explanation comes from the principle of stochastic resonance, whereby neural communication is enhanced by low levels of noise that can push a neural signal past threshold (Schwarzkopf et al., 2011; Silvanto and Cattaneo, 2017). Thus, 1 Hz rTMS may enhance episodic memory by interacting with intensity to enhance the signal-to-noise ratio in the stimulated network of neurons. However, this process is affected by a variety of other factors that further complicate the process. For example, the effects of MTI are known to be sensitive to “brain states” and changes in brain activity can influence if below- or above-MTI leads to facilitatory or inhibitory effects (Romei et al., 2016).

Stimulation intensity also interacted with 20 Hz rTMS with attenuating effects of below-MTI compared to at-MTI. These findings are not consistent with stochastic resonance, where below MTI stimulation should lead to enhancing effects. However, the situations in which different levels of intensity will lead to either enhancing or inhibitory effects have also been known to depend on brain states. One reason for the observed effects may be due to differences in experimental designs, as task stimuli or cognitive processes engaged likely resulted in different activation states in the targeted brain regions. Relatedly, previous work has shown that cortical excitability varies with different MTI levels following iTBS (Chung et al., 2018) protocols. Thus, it is possible that the enhancing or inhibitory effects of rTMS may vary with MTI, which may also differ depending on the frequency of stimulation.

### Interaction Between rTMS Frequency and Stimulation Timing

Additional analyses revealed that the effects of episodic memory performance in young adults depended on frequency and stimulation timing. Specifically, the enhancing effects of 1 Hz compared to 20 Hz was driven by online protocols. On the other hand, both 1 Hz and 20 Hz rTMS protocols had enhancing effects compared to either iTBS or cTBS paradigms when stimulation occurred offline. Interestingly, online 20 Hz rTMS protocols had inhibitory effects on episodic memory; however, offline 20 Hz rTMS had enhancing effects on episodic memory. These findings suggest that 20 Hz rTMS modulates episodic memory through different mechanisms when applied online vs. offline. For example, online paradigms may disrupt ongoing neural activity while offline paradigms may potentiate episodic memory processes through LTP-like effects.

Taken together, while the meta-analysis suggests novel perspectives on the effects of interacting factors on rTMS studies of episodic memory, the limited numbers of studies with combinations of factors spanning the entire parameter space may have hindered our ability to reliably estimate the role of many factors (e.g., targeted hemisphere, cortical region, memory process, and retrieval type) that are hypothesized to moderate the effects of rTMS on episodic memory. Moreover, the current meta-analysis did not find a moderating effect of the type of control condition (i.e., active, active vertex, sham, or no TMS) that was implemented. Importantly, these findings do *not* suggest that there are no differences between using active, vertex, sham, or no TMS control conditions. There are substantial gains in the quality of inference that can be obtained with comparison to proper control conditions. Inferences about the role of a particular brain region in a specific process are strengthened when effects are contrasted against conditions in which active stimulation at the same level of intensity and frequency is applied to either the targeted brain region at a different time point or to a different brain region that is not hypothesized to be involved in the process of interest at the time of stimulation. Therefore, researchers should still take careful consideration in the type of control condition that is most appropriate and feasible for specific experimental designs (for further information on control conditions see, Davis et al., 2013; Duecker and Sack, 2015).

In the next section we discuss how the meta-analysis on younger adults relates to the pattern of observed effects of rTMS effects on episodic memory functioning in healthy older adults and those with clinical disorders. This systematic review of the effects of rTMS on older adults revealed several similar findings that were observed in the meta-analysis on younger adults, thereby providing some sense of cross-validation of the findings in different populations.

## Comparisons to rTMS Effects on Older Adults and Clinical Populations

### Enhancing Effects of 1 Hz rTMS When Applied Below MTI

A similar pattern of effects of 1 Hz rTMS on episodic memory that was revealed in the meta-analysis on young adults was found in 1 Hz rTMS studies on older adults with below-MTI stimulation enhancing performance (Turriziani et al., 2012) while above-MTI had no effect (Davis et al., 2017). Turriziani et al. (2012) administered offline 1 Hz rTMS at 90% MT to the left or right DLPFC of patients with mild cognitive impairment following encoding of faces, buildings, and words and found enhanced recognition memory. In contrast, Davis et al. (2017) administered offline 1 or 5 Hz rTMS at 120% MT to the left DLPFC of healthy older adults prior to an encoding task and observed null effects on associative memory (although there were differential effects on brain network activity related to successful memory performance, for details see Davis et al., 2017). Although several parameters differed between these studies, the difference in outcomes of 1 Hz rTMS as a function of MTI is consistent with the findings of studies included in the meta-analysis irrespective of differences among those studies. However, there are not enough studies available to compare all combinations of parameters of offline 1 Hz rTMS below-, at-, or above-MTI stimulation.

### Impairments From Online 20 Hz rTMS Below MTI

Twenty Hz rTMS findings in healthy older adults found a similar pattern that was revealed in the meta-analysis with impairments to episodic memory when 20 Hz rTMS was administered online or below MTI. Rossi et al. (2004) reported attenuating effects of online 20 Hz rTMS at 90% MT to the left DLPFC during picture retrieval. In addition, Manenti et al. (2011) found attenuating effects on episodic memory when online 20 Hz rTMS at 90% RMT was applied to left vs. right DLPFC during either encoding or retrieval. However, this effect was largest in low performing adults when stimulation was applied to left compared to right DLPFC during encoding. Thus, it is possible that the effects of 20 Hz rTMS on episodic memory may rely on more complex interactions that were not possible to examine in the current meta-analysis due to the limited number of studies that have investigated the combinations of parameters.

### Enhancing Effects of Offline 20 Hz rTMS

Two studies on older adults with memory disorders found a similar pattern that was revealed in the meta-analysis with enhancing effects of multi-session administration of offline 20 Hz rTMS (Wang et al., 2014; Nilakantan et al., 2017). In prodromal Alzheimer's patients, Koch et al. (2018) reported that 10 sessions (5 sessions/week) with 2-week separation between cross over stimulation (active vs. sham) of offline 20 Hz rTMS at 100% RMT to the precuneus enhanced delayed recall (Koch et al., 2018). Similarly, in an individual with mild cognitive impairment, Cotelli et al. (2012) found enhanced associative memory compared to healthy controls following 10 sessions (5 sessions/week) of offline 20 Hz rTMS at 100% MT to the left parietal cortex (Cotelli et al., 2012).

Importantly, the enhancing effect of offline 20 Hz contrasts with an overall impairment of online 20 Hz rTMS on episodic memory (see Table 2). However, offline 20 Hz studies implemented multi-session protocols (e.g., 5 sessions/week for 2 weeks) while all nine online 20 Hz studies implemented single session protocols (see Table 1), so this confounds interpretation of the potential difference in the direction of effects of 20 Hz rTMS when applied offline vs. online. This also reveals a gap in the literature as few studies have implemented multi-session protocols with younger adults. Collectively, these findings are encouraging with regards to the potential for applying multi-session offline 20 Hz rTMS protocols to enhance episodic memory in older adults, even those with MCI or AD.

### Enhancing Effects of Offline 5 Hz rTMS Below MTI

Three studies implemented 5 Hz rTMS and found enhanced behavioral outcomes (Solé-Padullés et al., 2006; Peña-Gomez et al., 2012) or modulation of neural activity (Davis et al., 2017). In low performing older adults, Solé-Padullés et al. (2006) reported enhancing effects when offline 5 Hz rTMS at 80% MT was applied prior to encoding face-name associations. In addition, in carriers of a major genetic risk factor for Alzheimer's disease (Apolipoprotein ε4 allele; APOE), enhancing effects were reported following offline 5 Hz rTMS at 80% MT prior to a face-name encoding task (Peña-Gomez et al., 2012). In healthy older adults, Davis et al. (2017) reported modulated neural activity, but no differences in memory performance following offline 5 Hz rTMS at 120% MT prior to an associative encoding task. There are no studies on young adults that administered offline 5 Hz rTMS. This prevents making comparisons and highlights the need for future work to address this gap.

### Enhancing Effects of Offline 10 Hz rTMS

Five studies applied offline 10 Hz rTMS to older adults or clinical populations that included a wide age range and all but two found enhancing effects on episodic memory (see Table 1). The offline 10 Hz studies that found null effects used a different stimulation intensity than the others (Rektorova et al., 2005) or applied rTMS to motor cortex in fibromyalgia patients (Baudic et al., 2013). The studies that found enhancing effects of offline 10 Hz used stimulation intensities below- or above-MTI (Drumond Marra et al., 2015; Qiao et al., 2016; Kavanaugh et al., 2018). These findings diverged from the results of the meta-analysis that revealed a trend for impairments of online 10 Hz rTMS in younger adults. Differences in the stimulation timing between studies is one potential reason for the different effects; however, direct comparison is complicated because no studies to date have applied offline 10 Hz in young adults.

## Limitations of Conducting a Meta-Analysis on Available rTMS research

Although the systematic review and meta-analysis revealed some promising findings, a considerable amount of research is needed to more fully understand the roles of both specific aspects of the rTMS parameters (frequency, intensity, timing, hemisphere, cortical region, and control conditions) and design considerations (targeted memory process and retrieval type) on the effects on episodic memory functioning, as well as how these numerous factors may interact. Because there are not published studies for every pairwise combination of the rTMS parameters and design aspects of interest, there were many missing cells in the meta-analysis. Relatedly, because of missing data cells and small numbers of observed effects sizes for each possible comparison, we weren't able to investigate higher-order interactions.

### Effects on Objective vs. Subjective Aspects of Episodic Memory

It should also be noted that the current meta-analysis focused on accuracy as the outcome measure of episodic memory performance. We did not address the potential moderating effects of rTMS and study design parameters on other measures of episodic memory performance such as reaction times and confidence ratings. These are important issues to investigate in future research as the body of literature accumulates.

For example, while the meta-analysis on accuracy measures failed to reveal a main effect or interaction with the cortical region that was targeted or the memory process that was stimulated, based on the systematic review of the published studies, it seemed to be the case that several studies found that applying rTMS to regions of parietal cortex affected the subjective experience of remembering as evidenced by modulation of confidence ratings even in the absence of effects on objective measures of accuracy. For example, Ye et al. (2018) recorded participants playing an action-adventure video game and then applied offline 1 Hz rTMS at 110% AMT to the precuneus prior to snapshot images from the video game and then tested subjects' temporal order memory and confidence ratings. The authors found decreases in confidence ratings during retrieval following precuneus compared to vertex stimulation, despite no differences in memory performance (Ye et al., 2018).

In a similar fashion, Bonnì et al. (2015) applied offline cTBS at 100% MT to the precuneus, posterior parietal cortex, or vertex prior to associative (color-context) memory tests of studied object images (e.g., apple outlined in red). While there were no differences in hit rates among the conditions, there was a significant decrease in the number of source memory errors (e.g., correct judgment that the apple was outlined in red not green) that were made following precuneus stimulation (Bonnì et al., 2015). Relatedly, offline 20 Hz rTMS at 100% MT to the left angular gyrus prior to retrieval of previously encoded pictures led to decreased recognition relative to superior parietal lobe stimulation, and a decrease in recollective details (by altering response bias) compared to either superior parietal or vertex stimulation (Sestieri et al., 2013). Moreover, offline cTBS at 70% RMT to the left angular gyrus prior to encoding auditory stimuli reduced subjective confidence ratings for source memory while leaving free- and cued- recall of word pairs unaffected (Yazar et al., 2014). Taken together, it seems that rTMS to parietal regions tends to affect the subjective experience of remembering even when there are null effects on episodic memory accuracy. However, too few studies have been published to allow for meta-analysis of the effects of moderators of the size of effects on episodic memory. The same was true for other important measures of episodic memory performance such as reaction times. Future research should address these limitations.

### Effects on Encoding, Consolidation, and/or Retrieval

Another limitation has to do with the complication of interpreting the effects of rTMS on episodic memory due to the design of many studies, particularly studies with multiple rTMS sessions or offline rTMS, because it is difficult to know if the effects of rTMS are modulating encoding, consolidation, and/or retrieval processes. Relatedly, to our knowledge, only one study implemented rTMS to definitively target consolidation-based processes. In a pair of studies, Sandrini et al. (2013) investigated the role of right DLPFC in the reconsolidation of memories for studied words of objects by administering offline 1 Hz rTMS at 100% MT. The first experiment spanned 3 days. On day one, participants learned a list of words with contextual reminders (e.g., specific room and colored bag containing the words). On day two, participants were assigned to receive either rTMS to right DLPFC during a pure consolidation phase without reactivating the memories or rTMS to right DLPFC or the vertex before reactivating the memories in a contextual reminder reconsolidation phase. Finally, on day three, participants underwent a free recall task in which participants recalled as many words as possible. The findings revealed that rTMS prior to memory reactivation led to memory enhancements compared to the no reactivation and vertex control conditions. To examine if rTMS effects were specific to reconsolidation and rather than either encoding or retrieval processes, a follow up experiment used a similar procedure with the exception that memory was assessed 1 h after stimulation instead of the next day. Given that reconsolidation processes take several hours, finding no memory differences across the conditions after 1 h supported the conclusion that the DLPFC was specifically involved in reconsolidation processes (Sandrini et al., 2013). The use of multiple active control conditions further helped the researchers to isolate the effects to reconsolidation processes (as opposed to initial encoding or retrieval). This study demonstrates the unique feasibility of using rTMS to systematically manipulate (re)consolidation processes. However, because there is so little research that has investigated the effects of rTMS on consolidation processes, the current meta-analysis was restricted to studies that have investigated effects on encoding and retrieval stages. This highlights an area where future TMS research is needed.

### Variability in the Quality of Targeting Procedures

Another limitation of the current meta-analysis is the possibility that we may have underestimated the true effect of rTMS on episodic memory performance across conditions. The quality assessment revealed that a large number of studies targeted cortical regions using the international 10–20 electrode positioning system. This approach is imprecise and does not take into account inter-individual differences in the location of specific brain structures. Structurally or functionally guided TMS targeting using a neuronavigation system with each subject's structural and/or functional MRI can more precisely stimulate the targeted brain region of interest. Modeling work that has contrasted the variety of targeting methods has shown that larger TMS effects are obtained with individual fMRI-guided TMS neuronavigation when targeting parietal regions than targeting with the 10–20 EEG positioning system, which yielded smaller effect sizes (Sack et al., 2009). Power analyses revealed that a sample size of 47 was required to detect a significant effect when the 10–20 EEG positioning system is used for TMS targeting (Sack et al., 2009). Given that a large number of studies implemented the 10–20 targeting system and the selected studies had an average sample size of 24.19 in the meta-analysis, it is possible that the existing effects sizes available in the published literature underestimate the true effect of rTMS on episodic memory. Another important aspect to note is that only published effect sizes could be included in the meta-analysis and, therefore, excluding any unpublished null effects would obscure and overestimate the true effect of rTMS on episodic memory. Reassuringly, the steps we took to investigate a potential publication bias suggests that this was unlikely to be of major concern. However, it should be noted that absence of small-study biases does not prove the absence of publication bias, and further research is needed to properly address publication bias in multi-level models.

## Considerations for Future Research

### Personalized rTMS

Below we highlight and discuss potentially important factors that need to be systematically investigated over the next 30 years of TMS research and beyond. One pervasive issue in the field has been the highly variable findings across both behavioral and neural outcomes. Certain individuals may respond to a set of rTMS parameters, while other individuals may have null or negative effects to the same rTMS protocol. One possible solution to this heterogeneity in the response to rTMS is to implement more personalized rTMS parameters titrated to match each individual's physiological characteristics that likely underlie the variability between individuals. We have already noted the need to use structural and functional neuronavigated targeting procedures. Other characteristics that are likely to be important concern the titration of the frequency of stimulation to the precise frequency of endogenous oscillations of interest in targeted brain regions.

For example, in a pair of studies, Waldhauser et al. (2016) implemented a personalized rTMS-EEG design to examine if reactivation of sensory information is necessary for recognition of studied items and/or their studied context/source. In experiment one, participants intentionally encoded visual stimuli (i.e., objects) presented laterally (left and right) to a fixation cross; at test, participants made old/new judgments and source judgments about whether the item initially appeared on the left or right side of the screen while undergoing concurrent EEG recording. Identification of a time window (33.5–204.5 ms), frequency (17.5 Hz), and source (MNI coordinates ± 40, −78, 0) of oscillatory behavior for successful source memory judgments were obtained by examining the lateralization of alpha/beta activity during encoding and retrieval. In a second experiment using the same encoding/retrieval procedure they applied rTMS (17.5 Hz) to the occipital lobe (MNI coordinates ± 40, −78, 0) during retrieval from 33.5 to 204.5 ms after cue presentation (Waldhauser et al., 2016). Importantly, when rTMS was applied using the specific temporal, spatial, and frequency parameters related to the task demands for successful episodic retrieval, there was a decrease in source memory performance relative to control.

In a similar study, Hanslmayr et al. (2014) compared online rTMS-EEG at 90% RMT in the beta frequency range (18.7 Hz) to 10.7, 6.8 Hz, and sham stimulation to the left IFG during word encoding. On a subsequent free recall task, participants showed memory impairment following 18.7 Hz stimulation compared to sham. Critically, despite targeting the same task-dependent region, there were no differences in memory performance for 10.7 or 6.8 Hz stimulation compared to sham. At the neural level, the degree of beta entrainment following 18.7 Hz stimulation was correlated with memory impairment, with higher levels of beta entrainment leading to greater decrements in memory performance (Hanslmayr et al., 2014). The findings that artificial beta synchronization leads to memory impairment supports electrophysiology findings that alpha/beta desynchronization is associated with memory enhancement (Hanslmayr et al., 2016). Thus, the implementation of more nuanced rTMS frequencies affords the possibility to more effectively modulate episodic memory.

### Aspects of Episodic Memory

In this review, many of the studies that assessed the effects of rTMS on episodic memory involved spatial locations/associations with stimuli (e.g., object-location associations). While spatial context information is often one important element of episodic memory, there are other important aspects of episodic memories (e.g., object, temporal, social, and emotional information) that may result in dissociable effects of rTMS to different brain regions. Furthermore, it is possible that the type of episodic information (e.g., visual, auditory, multi-modal) may interact with different aspects of episodic memory (e.g., object, temporal, and spatial). Future research should consider these potentially important moderating variables when investigating the effects of rTMS on various aspects of episodic memory.

### Closed Loop rTMS

The majority of rTMS findings have focused on researching the brain as a “black box” in an open-loop manner, whereby a priori input stimulation parameters remain constant and one observes the corresponding output. Emerging evidence from multi-modal approaches has begun to clarify how stimulation parameters such as timing and frequency modulate brain-behavior relationships. This has also led to a shift in focus from mapping segregated brain regions to a more interactive-systems approach by attempting to understand how fast-changing patterns of neural activity interact with local and distal brain regions to give rise to human behavior. Understanding the frequency and temporally specific manner that different cortical regions interact has helped uncover how neural processing is linked with behavior. However, the optimal stimulation parameters that lead to episodic memory enhancement or detriment have not been clearly identified and the sparseness of available findings await replication and extension with systematic manipulations to map the parameter space. Moreover, there has been little consideration paid to how memory outcomes may be dependent on interactions between stimulation protocols and temporal patterns of initial brain state activity and ongoing neural activity during a task. The results of the current meta-analysis reveal that it is of paramount importance that future studies take this into consideration in order to determine optimal stimulation parameters for episodic memory (for additional information see, Bergmann et al., 2016)

While this “black box” approach has proven fruitful, the brain is a dynamic system capable of receiving, modulating, and generating both further inputs (modified representations, novel associations) as well as outputs (Berkman and Lieberman, 2011). A wave of technological advancements has made it possible to make stimulation adjustments based on real-time neural signals. This has given rise to an informed closed-loop paradigm where neural activity from EEG/MEG provide amplitude, phase, or power of oscillatory activity markers that allow for stimulation in a temporally specific fashion. This has also paved the way for fully adaptive closed-loops that aim to use stimulated neural markers to not only trigger stimulation but also to use neural activity to adaptively alter stimulation protocols in real time (Karabanov et al., 2016).

### Multi-Modal rTMS

Given the wealth of knowledge that has been obtained using multimodal approaches, extending these typically two-way combinations (e.g., TMS-EEG and TMS-fMRI) to three-way TMS-EEG-fMRI tools may provide a more holistic view of brain functioning. Such a combination with TMS enables novel stimulation findings based on the temporal precision of EEG to be complemented with fMRI to examine changes in functionally related neural activity in cortical and subcortical structures with high spatial resolution. The feasibility of combining TMS-EEG-fMRI has already been established to be safe and provide reliable signal recordings (Peters et al., 2013).

## Conclusion

The findings of the meta-analysis on younger adults revealed that frequency interacts with MTI as well as with stimulation timing. Specifically, both online and offline 1 Hz rTMS led to enhancing effects, which was driven by below-MTI stimulation. In addition, offline 20 Hz rTMS had enhancing effects whereas, online 20 Hz rTMS and 20 Hz rTMS at below-MTI led to impairing effects on episodic memory. A systematic review of the older adults and those with clinical disorders revealed a similar pattern of enhancing and attenuating effects of rTMS on episodic memory performance. However, important differences did arise with older adults and clinical populations implementing both offline 5 and 10 Hz rTMS protocols that had enhancing effects on episodic memory.

In sum, these findings highlight the importance of the contextual aspects of stimulation to reveal brain-behavior relationships at a more causal level than permitted by methods that are inherently correlational. Important contextual aspects to consider include the brain state of the targeted region and functionally connected regions at the time of stimulation. Furthermore, researchers must take into consideration the time-dependent processes engaged during specific encoding, consolidation, and retrieval phases that are coded by the brain region of interest, as well as variability both between and within individuals in the precise timing and magnitude of activation of such relevant brain areas. Multi-modal and closed-loop approaches offer promise for addressing these likely pernicious contributors to the variability that is seen across the literature, which may be necessary to parse out the role of exogenous stimulation of brain regions and functionally connected regions for modulating episodic memory. Although still in the initial stages of development, the future of closed-loop paradigms offers the ability to analyze stimulation induced brain activity to dynamically fine-tune stimulation parameters to facilitate rTMS effects on cognition. It is exciting to ponder what the next 30 years will elucidate regarding the use of non-invasive brain stimulation for causal modulation of brain-behavior relationships and, ideally, the enhancement of episodic memory in both healthy and clinical populations.

## Author Contributions

NY conducted the review and meta-analysis and wrote the initial drafts. NR helped design the study, provide edits, conceptual and theoretical feedback, and write the final draft.

### Conflict of Interest Statement

The authors declare that the research was conducted in the absence of any commercial or financial relationships that could be construed as a potential conflict of interest.

## Footnotes

^1^There was also a significant difference of 20 Hz rTMS “above” compared to both 20 Hz rTMS “at” and “below” MTI, although this is relatively uninformative given that only one study applied 20 Hz rTMS “above” MTI, see Tables 4 and 5.
